# Designing stable, hierarchical peptide fibers from block co-polypeptide sequences†
†Electronic supplementary information (ESI) available: Experimental information, peptide analysis, supplementary figures and tomography reconstruction videos. See DOI: 10.1039/c9sc00800d


**DOI:** 10.1039/c9sc00800d

**Published:** 2019-08-07

**Authors:** Mark M. J. van Rijt, Adriano Ciaffoni, Alessandro Ianiro, Mohammad-Amin Moradi, Aimee L. Boyle, Alexander Kros, Heiner Friedrich, Nico A. J. M. Sommerdijk, Joseph P. Patterson

**Affiliations:** a Laboratory of Materials and Interface Chemistry , Centre for Multiscale Electron Microscopy , Department of Chemical Engineering and Chemistry , Eindhoven University of Technology , P. O. Box 513 , 5600 MB Eindhoven , The Netherlands . Email: patters3@uci.edu ; Email: nico.sommerdijk@radboudumc.nl; b Institute for Complex Molecular Systems , Eindhoven University of Technology , P. O. Box 513 , 5600 MB Eindhoven , The Netherlands; c Department of Supramolecular & Biomaterials Chemistry , Leiden Institute of Chemistry , Leiden University , P. O. Box 9502, 2300 RA , Leiden , The Netherlands; d Laboratory of Physical Chemistry , Department of Chemical Engineering and Chemistry , Eindhoven University of Technology , P. O. Box 513 , 5600 MB Eindhoven , The Netherlands

## Abstract

Natural materials, such as collagen, can assemble with multiple levels of organization in solution. Achieving a similar degree of control over morphology, stability and hierarchical organization with equilibrium synthetic materials remains elusive. For the assembly of peptidic materials the process is controlled by a complex interplay between hydrophobic interactions, electrostatics and secondary structure formation. Consequently, fine tuning the thermodynamics and kinetics of assembly remains extremely challenging. Here, we synthesized a set of block co polypeptides with varying hydrophobicity and ability to form secondary structure. From this set we select a sequence with balanced interactions that results in the formation of high-aspect ratio thermodynamically favored nanotubes, stable between pH 2 and 12 and up to 80 °C. This stability permits their hierarchical assembly into bundled nanotube fibers by directing the pH and inducing complementary zwitterionic charge behavior. This block co-polypeptide design strategy, using defined sequences, provides a straightforward approach to creating complex hierarchical peptide-based assemblies with tunable interactions.

## Abstract 2

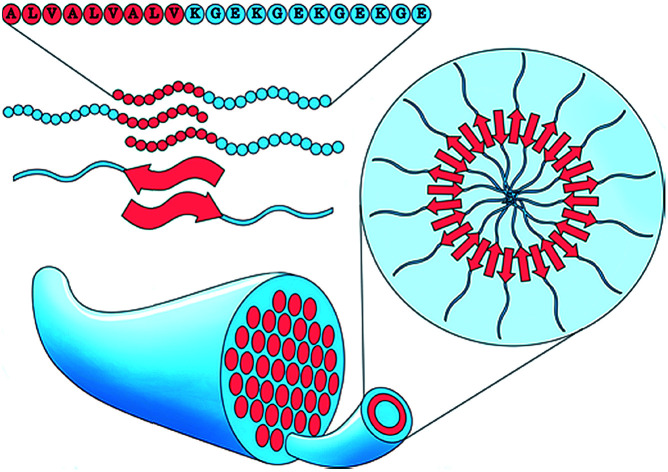
Here we report the pH induced self-assembly of equilibrium zwitterionically charged block co-polypeptide nanotubes into hierarchical nanotube fibers.

## Introduction

Due to their biocompatibility, biodegradability and their versatility in chemistry,^1^–^3^ poly(amino acid) amphiphiles are widely investigated for applications including therapeutics,^4^ drug delivery,^5^ and scaffolding for biological growth.^6^ A wide range of strategies exist for creating peptide based self-assembled materials including: dipeptides,^7^ dynamic peptide libraries,^8^ spider-silk based sequences,^9^ peptide amphiphiles (PA)^10^,^11^ and block co-polypeptides (BCPP).^12^,^13^ These amphiphilic peptide materials have been shown to organize into various morphologies, such as; spherical-,^14^–^17^ and cylindrical micelles,^6^,^8^,^18^ vesicles,^12^,^17^,^19^–^21^ nanotubes,^22^–^30^ nanoribbons,^25^,^30^–^34^ and hydrogel networks.^13^,^35^ Nanotubes are defined as well-defined hollow cylinders with a diameter range of 0.5–500 nm and an aspect exceeding five.^36^ These morphologies possess only limited levels of organization, which strongly contrast with natural materials like collagen that possess multiple levels of organization (hierarchical materials). As these hierarchical materials possess unrivalled control over structure and properties,^37^ achieving hierarchical self-assembly in synthetic materials through additional complementary supramolecular interactions is an important goal in the field of bio-inspired materials.

Synthetic hierarchical materials often form kinetically trapped structures, which can be very stable,^38^–^40^ but generally are highly dependent on the preparation conditions and mostly are disperse in size and morphology.^41^,^42^ Thermodynamically controlled assemblies that rapidly equilibrate to the lowest energy conformation tend to form well-defined and reproducible structures.^43^ However, these structures tend to rearrange upon changing solution conditions,^44^ which limits their usability window and prevents control over their organization in solution. At elevated temperatures, changes or even denaturation of the peptide secondary structure can radically influence the expressed morphology resulting in for example, peptide nanotube in helical unwinding,^29^ or the devolution into spherical micelles at elevated temperatures.^25^ The pH induced protonation or deprotonation of peptide moieties has shown to induce self-assembly,^45^ evolution in morphology,^18^ or even the inversion of vesicular assemblies.^19^ Furthermore, variation in ionic concentration have shown to both influence the secondary structure and the fibrillar length.^6^ Therefore, realizing thermodynamically controlled assemblies that are stable in a wide range of environments with the ability to form hierarchical assemblies is an interesting challenge which requires a careful balance of system thermodynamics and kinetics.^46^ For peptide-based assemblies this means controlling hydrophobic interactions, secondary structure and electrostatics.^11^,^47^ Consequently, new approaches towards designing peptide sequences which allow control over thermodynamics and kinetics are required.

Here we use BCPPs with a defined amino acid composition and sequence, inspired on previous observed assembly behavior.^48^ We designed a hydrophobic core sequence with the ability to form secondary structure, and a hydrophilic “stabilizer” sequence which is pH responsive. Simple variation of the relative block lengths and their ability to form secondary structure provides control over the system morphology, thermodynamics, and kinetics. This allows us to form well-defined peptide nanotubes which are stable between pH 2–12, in a temperature range of 4–80 °C and under a wide range of ionic strengths. The stability of the nanotube morphology under this broad variety of environments allows their organization in solution to be tuned by controlling inter-nanotube attraction and repulsion, resulting in the formation of bundled nanotube fibers.

## Results and discussion

### Molecular design strategy

**Scheme 1:**
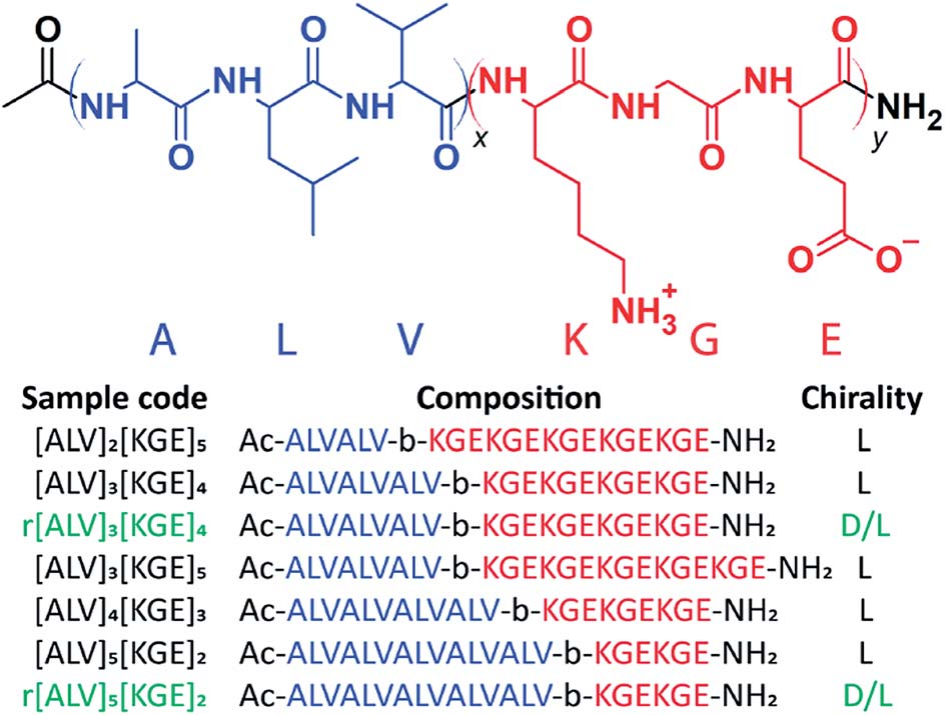
Molecular structure of [ALV]_*x*_[KGE]_*y*_ and an overview of the investigated peptide sequences.

Using solid-phase peptide synthesis (SPPS),^49^ we created a set of BCPPs with the generic composition [ALV]_*x*_[KGE]_*y*_, see Scheme 1 and ESI Section 3.† The hydrophobic alanine – leucine – valine (ALV) sequence is designed to form a rigid secondary structure, either α-helical or β-sheet,^50^,^51^ which makes the formation of typical spherical or cylindrical micelles less favorable as compared to the lower curvature nanotube or vesicular morphologies.^52^ The added rigidity of the secondary structure should also provide enhanced stability to the assembled morphology compared to typically used aliphatic segments. The alternating charges in the lysine – glycine – glutamic acid (KGE) hydrophilic sequence is designed to control the repulsion between individual chains and ultimately larger assemblies by adjusting solution pH. Based on the pH the hydrophilic stabilizer block is either dominantly positively, zwitterionically or negative charged. By varying the relative lengths of each sequence and synthesizing complementary racemic sequences, we investigate the relative contributions of secondary structure and hydrophobicity on the morphology and the thermodynamics and kinetics of the assemblies.

### Control over morphology and assembly kinetics by varying the hydrophobic–hydrophilic balance and secondary structure

To initiate self-assembly, the investigated peptide sequences (Scheme 1) were added directly to pure water (resulting in a pH of ∼4) or pH 4 buffer at a concentration of 5 or 10 mg mL^–1^. At both concentrations similar behavior was observed. For [ALV]_5_[KGE]_2_ this resulted in macroscopic phase separation. Assembly of [ALV]_5_[KGE]_2_ could be induced by a DMSO solvent switch procedure (see ESI Section 1†), still yielding a turbid dispersion. The high water-incompatibility of [ALV]_5_[KGE]_2_ indicates the formation of kinetically trapped assemblies during water addition in the solvent switch procedure.^53^ Upon pure water or pH 4 buffer addition the more hydrophilic sequences [ALV]_2_[KGE]_5_, [ALV]_3_[KGE]_4_, [ALV]_3_[KGE]_5_ and [ALV]_4_[KGE]_3_ quickly formed slightly viscous transparent solutions.

**Fig. 1:**
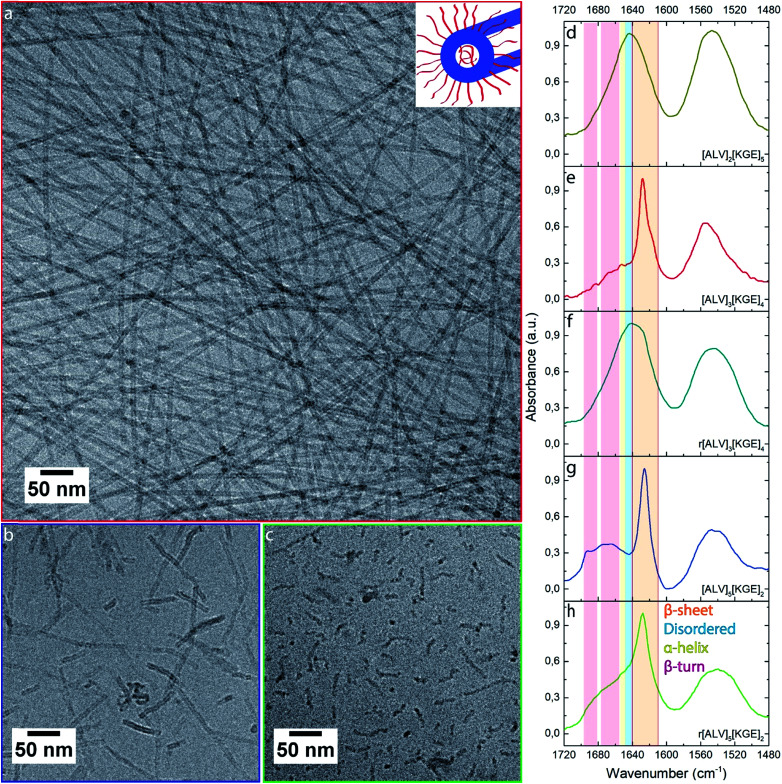
CryoTEM images (a–c) of [ALV]_3_[KGE]_4_ (a), [ALV]_5_[KGE]_2_ (b) and r[ALV]_5_[KGE]_2_ (c) combined with normalized FTIR spectra (d–h) of [ALV]_2_[ KGE]_5_ (d), [ALV]_3_[KGE]_4_ (e), r[ALV]_3_[KGE]_4_ (f), [ALV]_5_[KGE]_2_ (g) and r[ALV]_5_[KGE]_2_ (h) self-assembled at 5 mg mL^–1^. The FTIR spectra show the amide I and II signals of the peptide assemblies. Inset (a) corresponds to a sketch of the expected tubular assembly structure based on cryoTEM observations. Low magnification images of a-c can be found in ESI Section 4.†

**Fig. 2:**
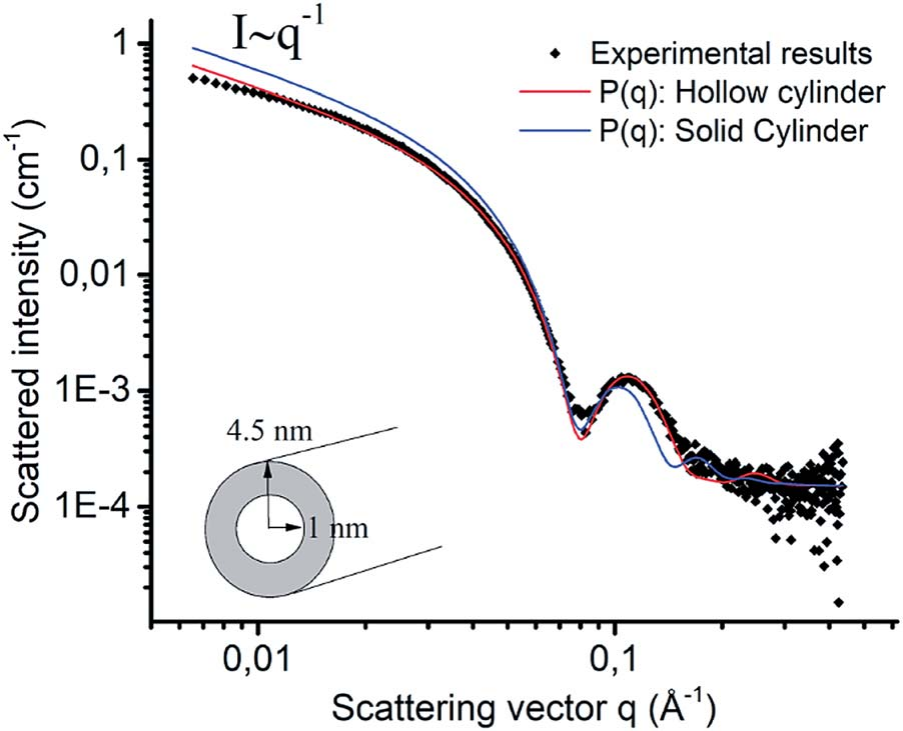
SAXS scattered intensity plot against the scattering vector (i) for [ALV]_3_[KGE]_4_ assembled at 5 mg mL^–1^ in pH 4 buffer by direct dissolution (black) *vs.* the fitted model for solid (blue) and hollow (red) cylindrical shaped micelles.

The evolution of morphology with increasing length of the hydrophobic sequence was investigated by cryoTEM. [ALV]_2_[KGE]_5_, the most hydrophilic sequence, showed no evidence of self-assembly (Fig. S1a†). Using cryo-EELS analysis a significant amount of nitrogen could be detected in the solution (Fig. S1c†), indicating that [ALV]_2_[KGE]_5_ peptides are solubilized as unimers, *i.e.* individual macromolecules. The presence of the peptide in solution was further supported by the formation of sheet-like polymer structures upon *in-microscope* freeze drying (Fig. S2a and b†). For the [ALV]_3_[KGE]_4_ peptide, the formation of well-defined nanotubes, with lengths of >2 μm and diameters of 9 ± 1 nm and with an internal cavity of 4.5 ± 0.4 nm were observed by cryoTEM (Fig. 1a). This measured diameter exceeds the length of two fully extended hydrophobic sequences (6.9 nm, ESI Section 5†), thereby strongly suggesting that the observed structures are hollow nanotubes (curled bilayer structures) rather than solid or hydrated cylindrical micelles. However, the observation of these small internal nanotube cavities by cryoTEM was very challenging and required optimal imaging conditions, as further discussed in ESI Section 5.† SAXS measurements were performed to support the cryoTEM results. The experimental SAXS data was compared to both form factor modelling (no least-squares minimization) and fitting (using least-squares minimization) of hollow and solid cylinders (Fig. 2, ESI Section 5†). For both procedures the solid cylinder model showed a poor correlation with the experimental results. In contrast, good agreement between the SAXS data and the hollow cylinder model, with a diameter of 9 nm with an internal cavity diameter of 2 nm, is observed, confirming the formation of nanotubes. That this internal cavity shows a smaller diameter according to SAXS (2 nm) compared to cryoTEM (4.5 nm) further supports the presence of hydrated stabilizer blocks in the nanotube interior. These hydrated domains cannot be observed by cryoTEM whereas they seem to provide contrast in SAXS.

To determine if these nanotubes are equilibrium species, dialysis was used, in combination with UV-Vis measurements of the amide absorption (ESI Section 6†), where the use of a 10 kDa dialysis membrane should only allow the diffusion of peptide unimers through the membrane wall. After 72 h of dialysis a strong decrease in amide absorption was observed, suggesting that unimer exchange between the nanotubes and bulk solution indeed occurs, strongly indicating that the nanotubes are equilibrium species.^54^ CryoTEM showed that similar tubular assemblies were found when [ALV]_3_[KGE]_4_ was subjected to DMSO solvent switch (ESI Section 4†). As the observation of similar structures from a different preparation method is uncommon for kinetically trapped structures,^54^ this lends support to our conclusion that these are in equilibrium with unimers in solution.

To determine if the evolution from unimers to nanotubes between [ALV]_2_[KGE]_5_ and [ALV]_3_[KGE]_4_ is due to an increase of the hydrophobic (ALV) sequence, or a decrease of the hydrophilic (KGE) sequence length, [ALV]_3_[KGE]_5_ was synthesized. CryoTEM showed identical nanotube assemblies compared to [ALV]_3_[KGE]_4_ (Fig. S3a†). This suggests that the increase in hydrophobic domain length is the driving force for the observed evolution in morphology.

Assembly of the [ALV]_4_[KGE]_3_ sequence resulted in a mixture of disordered curved cylindrical shaped micelles with a diameter of 5 ± 1 nm (Fig. S3b†) and long nanotubes with a diameter of 9 ± 1 nm. The latter had an internal cavity of 2.5 ± 0.4 nm. The assembly of [ALV]_4_[KGE]_3_ in two distinct cylindrical populations suggest the presence of a kinetic component during self-assembly.

CryoTEM further showed that the most hydrophobic peptide [ALV]_5_[KGE]_2_, assembled into 200 nm long nanotubes with a diameter of 9 ± 1 nm (Fig. 1b) and an internal cavity of 4.4 ± 0.4 nm. Based on the previously mentioned water-incompatibility of [ALV]_5_[KGE]_2_ we conclude that these nanotubes are kinetically trapped structures. This evolution from (1) soluble unimers of [ALV]_5_[KGE]_2_, to (2) nanotubes of both [ALV]_3_[KGE]_4_ and [ALV]_3_[KGE]_5_ that are in equilibrium with the solution, to (3) kinetically trapped structures with [ALV]_4_[KGE]_3_ and finally (4) phase separation of [ALV]_5_[KGE]_2_ (which can be assembled by solvent switch into kinetically trapped nanotubes) indicates that both the morphology and the energetics of the formation pathways can be controlled by the relative block lengths of the [ALV]_*x*_[KGE]_*y*_ system.

For all assembled samples, strong light scattering of the aggregates prevented the use of circular dichroism, therefore the secondary structure (*i.e.* folding behavior) of these macromolecules was investigated using Fourier transform infrared (FTIR) spectroscopy, by analyzing the amide I vibrations (Fig. 1d–h and S3c†). Hand-in-hand with the observed evolution in morphology, an evolution in secondary structure was observed: [ALV]_2_[KGE]_5_ showed an amide I maximum at 1644 cm^–1^ corresponding to disordered folding behavior; [ALV]_3_[KGE]_4_, [ALV]_3_[KGE]_5_ and [ALV]_4_[KGE]_3_ showed a dominant β-sheet folding behavior represented by an amide I maximum at 1627 cm^–1^; while [ALV]_5_[KGE]_2_ showed an amide I maxima at 1626 cm^–1^ and a mixed signal between 1665 cm^–1^, and 1693 cm^–1^ corresponding to a mix of β-sheet and β-turn folding behavior.^55^

To identify the influence of secondary structure both on morphology and equilibrium behavior of the assemblies we synthesized the racemically randomized peptides r[ALV]_3_[KGE]_4_ and r[ALV]_5_[KGE]_2_. Both could be dispersed by direct dissolution. Similar to [ALV]_3_[KGE]_4_, r[ALV]_3_[KGE]_4_ quickly formed a slightly viscous transparent solution. However, where the optically pure [ALV]_3_[KGE]_4_ formed well-defined nanotubes, cryoTEM analysis showed virtually no self-assembly for r[ALV]_3_[KGE]_4_ (Fig. S1b†), with exception of some small populations of cylindrical shaped micelles (Fig. S4†). After *in-microscope* freeze drying large amounts of micron-sized polymer sheets were observed (Fig. S2c and d†), that were not observed previously by cryoTEM or by dispersion turbidity. This indicates they were formed during freeze-drying and that most of the peptide was present in solution as unimers. For FTIR of r[ALV]_3_[KGE]_4_ (Fig. 1f and S5†) the amide I maximum was observed at 1642 cm^–1^ corresponding to disordered folding behavior, similar to that observed for [ALV]_2_[KGE]_5_.

The more hydrophobic sequence r[ALV]_5_[KGE]_2_ required multiple hours of stirring to obtain a transparent solution, but in contrast to the optically pure [ALV]_5_[KGE]_2_, it could still be dispersed by direct dissolution. r[ALV]_5_[KGE]_2_ showed the formation of disordered curved cylindrical shaped micelles with a diameter of 7 ± 1 nm (Fig. 1c). No internal cavities could be observed by cryoTEM, suggesting that cylindrical micelles are formed instead of nanotubes. This is supported by the measured decrease in diameter from 9 to 7 nm compared to [ALV]_5_[KGE]_2_ (Fig. S6†). This diameter is reasonable for cylindrical micelles formation based hydrophobic sequence length (ESI Section 5†). FTIR (Fig. 1h and S5†) showed an amide I maximum at 1627 cm^–1^ with a broad shoulder towards the higher wavenumbers.^55^ This suggests a combination of β-sheet and disordered folding behavior after self-assembly, and hence that β-sheet formation is an important stabilizing factor for the nanotubes.

The observation that these two racemic systems behave distinctly different from their optically active counterparts strongly suggests that the rigid β-sheet core folding is responsible for directing the nanotube structure in [ALV]_3_[KGE]_4_, [ALV]_3_[KGE]_5_, [ALV]_4_[KGE]_3_ and [ALV]_5_[KGE]_2_. At the same time β-sheet formation reduces the solubility of [ALV]_5_[KGE]_2_ compared to r[ALV]_5_[KGE]_2_ such that direct dissolution is not possible, and also unimer exchange is disfavoured.^44^

The above results stress the fine balance in composition required for the formation of equilibrium nanotube structures in water in the [ALV]_3_[KGE]_4_ system, making this peptide system an ideal candidate for studying the stability and responsive behavior of peptide aggregates.

### Thermal stability and thermodynamic control

To investigate if the [ALV]_3_[KGE]_4_ nanotubes are in equilibrium with the solution we performed controlled heating and cooling (thermal annealing) experiments monitoring structure formation with static light scattering (SLS) measurements. CryoTEM studies at room temperature before and after thermal annealing and for a reheated part of the sample to 58 °C (equipment limit) during vitrification were used to provide further insight into the system behavior.

During each heating step the total light scattering intensity at 90° was measured to determine the equilibration time. This showed that during every step full system equilibration occurred within 80 seconds after reaching the target temperature (Fig. S7†). At room temperature SLS shows that the logarithm of the excess Rayleigh ratio log_10_ *R*(*q*) scales linearly with the logarithm of the scattering vector log_10_ *q*, with a slope close to –1, corresponding to the presence of cylindrical shaped assemblies or nanotubes with a fractal dimension of 1.^56^

**Fig. 3:**
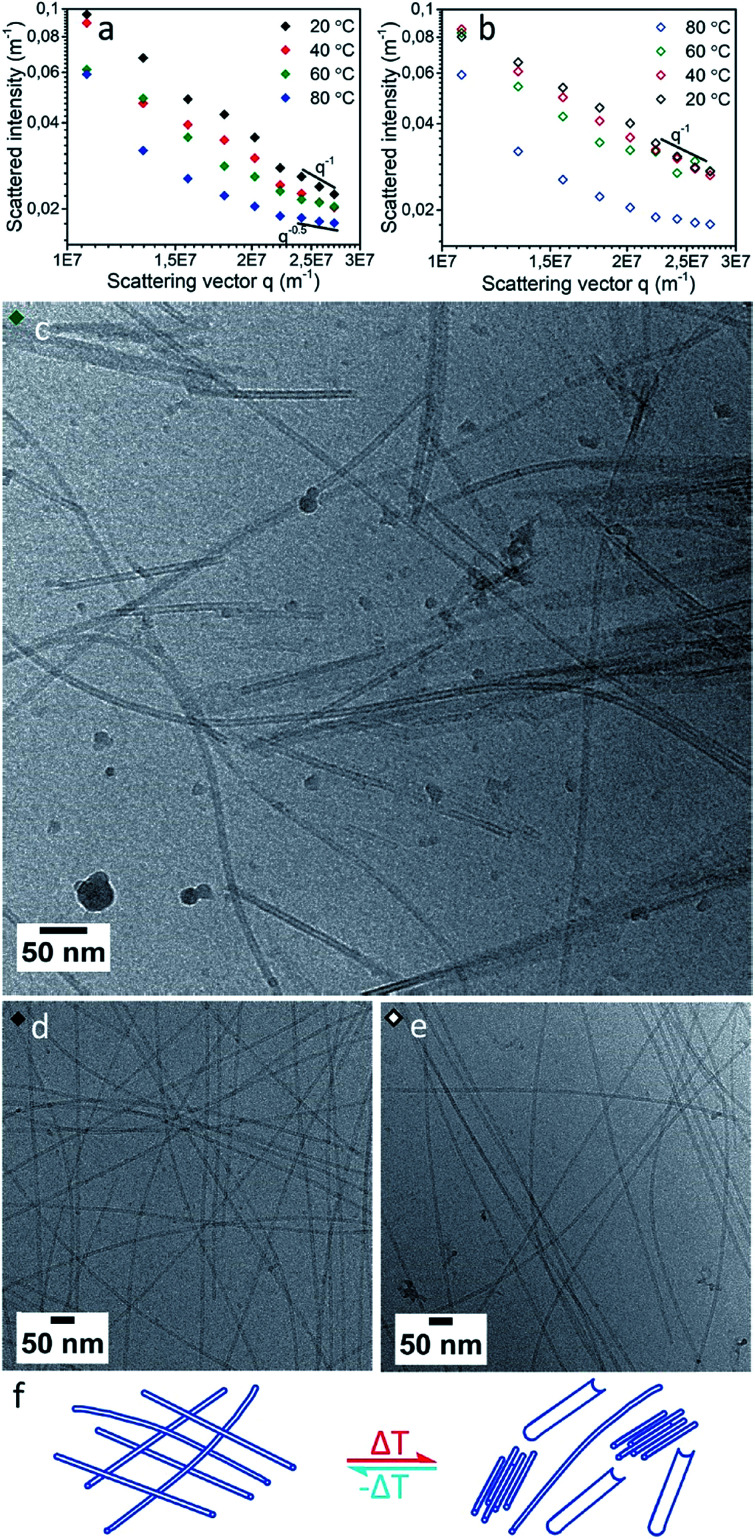
SLS results for [ALV]_3_[KGE]_4_ at 0.5 mg mL^–1^ in pH 4 buffer collected at 20, 40, 60 and 80 °C, during heating (a) and cooling (b). CryoTEM of [ALV]_3_[KGE]_4_ heated to 58 °C during vitrification (c), before heating (d) and after the SLS heating procedure (e). Scheme of observed species (f) before/after heating (left) and during at ∼60 °C (right).

With increasing temperature, the scattering intensity decreased (Fig. 3a) indicating a progressive decrease in nanotube length with temperature. Simultaneously, we observed an increase in the slope at low *q* values (<2 × 10^7^ m^–1^), indicating a progressive increase in attractive interactions between individual cylinders with increasing temperature. This strongly implies a tendency of the cylinders to form bundles at high temperatures. This trend is observed with a single exception at 60 °C at a *q* of 1.1 × 10^7^ m^–1^, likely due to the presence of intermediate aggregates. At 58 °C cryoTEM indeed showed the presence of large populations of bundles of relative short nanotubes. These bundles were present alongside a minor population of long (>2000 nm) curved nanotubes and some sheet like structures which possibly resulted from the unfolding of nanotubes (Fig. 3c and S8†).

In contrast, during gradual cooling from 80 to 20 °C the SLS curve (Fig. 3b) did not show a gradual transition. Instead, the decrease in intensity showed a strong hysteresis, although the curve recorded at 20 °C again matched that of the sample before heating, the observed hysteresis suggests that although the nanotubes show fully reversible aggregation behavior, the kinetics of aggregation are different from those of the de-aggregation process.^3^,^57^ CryoTEM showed similar long nanotube assemblies both before and after thermal annealing (Fig. 3d and e), and after thermal annealing unfolded nanotubes were no longer observed. This confirms that the SLS observations are indeed in accord with a fully recovered morphology and suggests that long nanotubes are the thermodynamically favored product.^6^

At elevated temperatures peptide nanotubes tend to morphologically rearrange.^25^,^29^ For [ALV]_3_[KGE]_4_, both SLS and cryoTEM suggest that the nanotube morphology is conserved even at high temperatures. The decrease in tube length can be explained by a shift in the thermodynamic equilibrium between unimers and assemblies at higher temperatures, and the concomitant dissolution of the unimers from the nanotube ends that have higher surface energies.^58^ The additional formation of bundled structures indicates an increase in interaction between individual nanotubes. This is likely due to thermal dehydration of the hydrogen-bonding groups in the hydrophilic stabilizer blocks, decreasing their solubility and leading to the loss of the hydration layer around the individual nanotubes, making lateral aggregation more favorable. Evidence for dehydration was indeed observed by cryoTEM which showed that at 58 °C (Fig. 3c) the nanotube wall gave a higher contrast compared to RT observations (Fig. 3d and e). The increase in nanotube wall density is consistent with the expulsion of water from the hydrophilic stabilizer block. Together SLS and cryoTEM suggest [ALV]_3_[KGE]_4_ nanotubes show significant temperature stability and form under thermodynamic control.

### pH responsive behavior and hierarchical assembly

The [ALV]_*x*_[KGE]_*y*_ peptides are designed with a complementary zwitterionically-charged hydrophilic stabilizer possessing alternating positive and negative charges. This should ensure stability of the assemblies at both low and high pH, where the stabilizer blocks will be positively charged or negatively charged, respectively, while promoting interactions between assemblies at intermediate pH. To investigate nanotube stability over a range of pH values, and to investigate whether pH modulation can activate the formation of higher order species without compromising the underlying nanotube morphology, [ALV]_3_[KGE]_4_ was assembled in buffers of pH 2, 6 and 12 (10 mg mL^–1^, direct dissolution). FTIR showed the formation of dominant β-sheet arrangements in all cases with amide I maxima between 1628–1624 cm^–1^ (Fig. S9†), suggesting a core folding similar to beta sheet formation observed at pH 4.

**Fig. 4:**
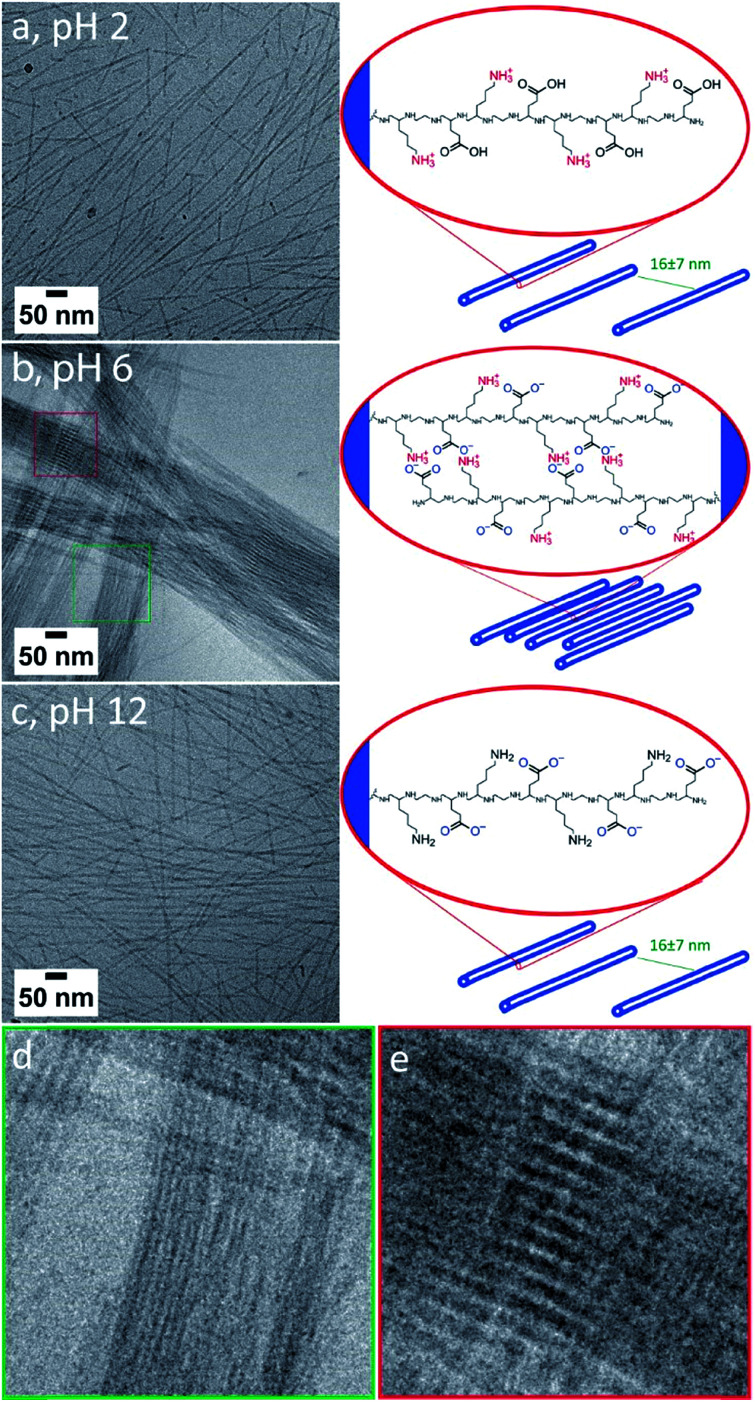
CryoTEM images and schemes of [ALV]_3_[KGE]_4_ assembled *via* direct dissolution at 10 mg mL^–1^ in pH 2 (a), 6 (b) and 12 (c) buffer. Zoom-in of (b) showing the bundled composition out of nanotubes (d) and an organization-induced interference pattern (e).

At both pH 2 and pH 12 (Fig. 4a and c) cryoTEM showed the formation of straight dispersed nanotubes with an external diameter of 8 ± 1 and 9 ± 1 nm, respectively, indicating that the nanotube morphology is highly stable over a wide range of pH values. The length of the nanotubes appears to vary with pH (Fig. 1a, 4b and c), where more significant populations of short nanotubes are present at pH 2 and 12. As the energy difference between long and short nanotubes will be small (aspect ratios >20) it is likely that the distribution of lengths is determined stochastically by the nanotube nucleation rate, which is likely pH dependent. At pH 6 the system becomes rather viscous and turbid, suggesting the presence of larger assemblies. CryoTEM in combination with cryogenic electron tomography (CryoET) indeed revealed the formation of large fibers of closely packed nanotubes (Fig. 4b and S11†). CryoET specifically confirmed that the fibers are three-dimensional structures composed of individual nanotubes with a diameter of 9 ± 1 nm (Fig. S10, ESI Videos 1 and 2†). Although the resolution of the reconstructions was not sufficient to observe the inner cavity of the nanotubes, their hollow nature could be confirmed from cryoTEM images (Fig. 4d and S11†). The conservation of the nanotube morphology shows that the β-sheet secondary structure provides strong enough intermolecular interactions that are not affected by the changed charge behavior of the hydrophilic stabilizer block. Most fibers were relatively thin (composed of <30 nanotubes per fiber) yet highly ordered as observed as interference patterns in cryoTEM (Fig. 4e). We propose that this order originates from the attractive forces between neighboring nanotubes that in combination with steric interactions induce their parallel alignment.

To substantiate this we consider the p*K*_a_s of the amino acids constituting the hydrophilic stabilizer blocks. Glutamic acid and lysine have p*K*_a_s of 4.25 and 10.53, respectively.^59^ At pH 6 both are expected to be charged, leading to zwitterionic peptide chains with strong mutual interactions. Diluting fibers formed at 10 mg mL^–1^ at pH 4 to 5 mg mL^–1^ at pH 6 results in the formation of high density nanotube patches in which nanotube alignment is preserved despite the dilution (Fig. S12a and b†). *Note: only dispersed nanotubes are observed when samples are prepared at 1 mg mL*^*–1*^*(see ESI Section 4†)*. However, when increasing the pH from 6 to 8, to a regime in which the lysine groups start to be deprotonated the alignment is lost and the fibers reorganize to form dispersed nanotubes, similar to those prepared directly at pH 8 (ESI Section 4, Fig. S12c†).

Hence, fiber formation appears to be controlled by electrostatic interactions between the hydrophilic stabilizer blocks. At pH 6 these will be in a zwitterionic state, allowing their interdigitation as proposed by Chen *et al.*^18^ Indeed, cryotomography shows that within the fibers the inter-nanotube distance is 2.5 ± 0.8 nm, which is significantly shorter than the length of a fully extended hydrophilic stabilizer sequence (4.4 nm), and supports the proposed interdigitation of the hydrophilic sequences as the driving force for fiber formation.

## Conclusions

Controlling thermodynamics and kinetics of molecular self-assembly to design objects with predesigned morphology and hierarchical structure is a key challenge for the creation of soft and complex materials. Here, we achieved this by the variation of the number and type of the amino acids in the hydrophilic and hydrophobic blocks of a block co-poly peptide. We demonstrated that by composing the appropriate hydrophobic core and hydrophilic stabilizer blocks we can create well-defined and thermodynamically stable nanotubes that can reversible assemble into fibers as a function of pH.

Varying the amino acid composition of the different blocks allowed us – beyond tuning the hydrophobic/hydrophilic balance – to modulate two parameters that were key to the assembly of these hierarchical structures: (1) the introduction of secondary structure (beta sheets) in the hydrophobic block, that provides the nanotubes with the required stability under different self-assembly conditions, (2) the reversible introduction of a zwitterionic regime in the hydrophilic blocks that allowed to direct the inter-nanotube interactions through pH variation.

Importantly, the thermodynamic stability of the nanotubes is a key factor in uniformity of the nanotube formation process, which, together with their high aspect ratios make this system an ideal candidate for further investigation as a peptide hydrogel system.^52^ Moreover, we anticipate that our approach can be used to design and control the thermodynamics, kinetics and morphology of peptide based assemblies for a range of applications.

## Funding sources

This project received funding from the 4TU High-Tech Materials research program “New Horizons in designer materials”, the Marie Sklodowska-Curie Action project “LPEMM” and from the Netherlands Organization for Scientific Research (NWO, TOP-PUNT Grant “Bi-Hy”, NWO-VENI and NWO-VICI project no. 724.014.001).

## Conflicts of interest

There are no conflicts to declare.

## Supplementary Material

Supplementary movie

Supplementary movie

Supplementary information
