# Supplementary material for: Designing stable, hierarchical peptide fibers from block co-polypeptide sequences
Source: Chem Sci. 2019 Aug 7;10(39):9001–8. doi: 10.1039/c9sc00800d (PMC7449534; doi:10.1039/c9sc00800d)
Supplement: Supplementary file 3 [file SC-010-C9SC00800D-s003.pdf]

Electronic Supplementary Material (ESI) for Chemical Science.
This journal is © The Royal Society of Chemistry 2019

Electronic supplementary information

Designing stable, hierarchical peptide fibers from block co-polypeptide sequences

Mark M. J. van Rijt[a,b], Adriano Ciaffoni[c], Alessandro Ianiro[b,d], Mohammad-Amin Moradi[a,b], Aimee L. Boyle[c], Alexander Kros[c], Heiner Friedrich[a,b], Nico A. J. M. Sommerdijk[a,b]* and Joseph P. Patterson[a,b]*

[a] Laboratory of Materials and Interface Chemistry & Centre for Multiscale Electron Microscopy Department of Chemical Engineering and Chemistry, Eindhoven University of Technology, P.O. Box 513, 5600 MB Eindhoven, The Netherlands

[b] Institute for Complex Molecular Systems, Eindhoven University of Technology, P.O. Box 513, 5600 MB Eindhoven, The Netherlands

[c] Department of Supramolecular & Biomaterials Chemistry, Leiden Institute of Chemistry, Leiden University, P.O. Box 9502, 2300 RA, Leiden, The Netherlands

[d] Laboratory of Physical Chemistry, Department of Chemical Engineering and Chemistry, Eindhoven University of Technology, P.O. Box 513, 5600 MB Eindhoven, The Netherlands

TABLE OF CONTENT

SI1 Experimental S2
- Materials S2
- Methods S3

SI2 Support figures S6

SI3 LC-MS data after purification S13

SI4 CryoTEM overview of assemblies S16

SI5 Discussion on nanotube formation S22
- Observance of hollow assemblies by cryoTEM S22
- Peptide molecular configuration S23
- Evidence of nanotube formation with SAXS S23

SI6 Dynamic self-assembly S25

REFERENCES S26

SI1 EXPERIMENTAL

Materials

All chemicals were used as received unless stated otherwise.

The peptides were synthesized by solid phase peptide synthesis (SPPS) using either a Liberty 1 or a Liberty Blue microwave-assisted automated peptide synthesizer. Peptides were synthesized on either ChemMatrix Rink amide resin (if the Liberty 1 was used) or TentaGel Rink amide resin (if the Liberty Blue was used). Standard Fmoc chemistry was employed, with deprotection facilitated by 20% piperidine in DMF and coupling achieved using either HCTU with DIPEA as base (with the Liberty 1) or DIC with Oxyma as base (Liberty Blue). Peptides were manually acetylated upon completion of synthesis using acetic anhydride and pyridine, before cleavage from the resin was facilitated using a mixture of TFA:TIPS:H_2O (95:2.5:2.5). The cleaved peptide was collected by precipitating into ice-cold diethyl ether, this was centrifuged and the supernatant was removed. The pellet was resuspended in a mixture of MeCN and H_2O before being freeze-dried.

pH buffers were prepared as shown in Table S1.[1]

Table S1 Measured pH and composition of used pH buffers.

pH		*M*	*A*	*ml*	*M*	*B*	*ml*	*Salt (mM)*
Target	*Measured*							
2	1.6	0.1	KCl	50	0.1	HCl	13	79.4
4	3.9	0.1	Acetic acid	164	0.1	CH_3COONa	36	72
6	5.9	0.1	KH_2PO_4	100	0.1	NaOH	11.6	89.6
8	7.9	0.1	KH_2PO_4	100	0.1	NaOH	93.4	51.7
10	9.8	0.05	$NaHCO_3$	100	0.1	NaOH	21.4	41.2
12	12.4	0.2	KCl	50	0.2	NaOH	12	161.3

Methods

High-performance liquid chromatography (HPLC) was conducted using a Shimadzu system with two LC-8A pumps and an SPD-10AVP UV-Vis detector. All peptides were purified using a Gemini 3 µm C18 column (150 x 21.2 mm). Peptides were eluted using a gradient of 10-90% B over 30 minutes at a flow rate of 12 mL min^{-1}, where A is H_2O containing 0.1% TFA, and B is MeCN containing 0.1% TFA. Collected fractions were analyzed using LC-MS and the fractions deemed to be >95% pure were pooled and freeze-dried.

Liquid chromatography mass spectrometry (LC-MS) was conducted on a Thermo Scientific LC-MS equipped with a Gemini® 3 µm C18 LC column (50 x 4.6 mm). MS and relative abundance measurements were conducted using a TSQ Quantum Access MAX MS chamber.

Self-assembly by direct dissolution (DD) was performed unless stated otherwise. Typically, $\leq$100 µl of purewater or buffer was added directly to the peptide powder, followed by overnight stirring using a magnetic stirring bar.

When mentioned, 50 v% diluted systems were made by adding $\leq$30 µl of the assembled peptide system with $\leq$30 µl dilutant followed by overnight stirring.

Self-assembly by solvent switch (SS) in DMSO was performed by dissolving peptide powder at 2 times the target concentration in DMSO. Pure-water was added dropwise under heavy stirring giving the system time to stabilize. DMSO was removed by dialysis using a Thermoscientific Slide-A-Lyzer® 2 kDa mini dialysis unit.

pH measurements were conducted using a Mettler Toledo seven compact pH/ion sensor or a Metrohm (6.0234.100) 125 mm pH probe.

Cryogenic transmission electron microscopy (Cryo-TEM) samples were prepared by depositing 3 µl sample on a 200 mesh Cu grids with Quantifoil R 2/2 holey carbon films (Quantifoil Micro Tools GmbH). An automated vitrification robot (FEI Vitrobot Mark III) was used for plunging in liquid ethane. All TEM grids were surface plasma treated for 40 seconds using a Cressington 208 carbon coater prior to use.

Cryo-TEM studies were performed on the TU/e cryoTITAN (FEI, www.cryotem.nl) operated at 300 kV, equipped with a field emission gun (FEG), a post-column Gatan Energy Filter (GIF) and a post-GIF 2k x 2k Gatan CCD camera.

Freeze-drying is preformed to confirm the presence of peptide on the grid, if no structures are observed during cryoTEM. Either these structures are removed during blotting or the peptides are present in unimer form. During freeze-drying, present peptides crash out of solution resulting in typically dense fibrous networks or sheets that can be observed by TEM. Freeze-drying is conducted in-microscope by gradually heating the samples on the grid in the autoloader to room temperature conditions overnight.

TEM image size analysis have been conducted using an in-house Matlab script. Rod diameters are estimated based on ideally 60 measurements from three representative micrographs (3 x 20). Clearly distinguishable cross sections are selected and measured at random. Where possible separate assemblies are measured targeting local maxima and minima. The interspacing distance of aligned rods determined,

by averaging the distances between the lowest intensity of the typical nanotube W-shape from intensity line plots gathered from at least three representative micrographs. The lengths of directional rods are estimated based on 10 end-to-end length measurements from a single representative micrograph. A length of > 2000 or > 1000 nm is indicated if respectively no rod ends or a single rod end is typically observed.

Cryogenic electron energy loss spectroscopy (Cryo-EELS) was conducted on the TU/e cryoTITAN in EELS mode at 390 eV with a width of 20 eV. Grid-carbon was avoided were possible. Spectra were collected between 3s and 10s. Beam damage was investigated by taking an image before and after EELS. EELS is used to identify present elements by looking at the loss energy of registered electrons. For nitrogen, which is only present in the Ac-$(ALV)_x$-b-$(KGE)_y$-NH_2 peptides the k loss edge is at 401 eV.

Cryogenic tomography series were collected between ± 66° using 3° increments, with a total electron dose below 60 $e^-/Å^2$. Tilt series acquisition was performed with Inspect3D software (FEI Company). Alignment and reconstruction of the series were carried out using IMOD software.

Attenuated total reflectance Fourier transform infrared spectroscopy (ATR-FTIR) measurements were conducted on a Varian 670-IR spectrometer using golden gate MCT setup taking 100 scans with a resolution of either 2 or 4 cm^{-1} between 4000 and 650 cm^{-1}. A 2 µl sample droplet was placed over the crystal without closing the golden gate. The environmental signals were deconvoluted from the peptide signals by subtracting a reference measurement.

Small angle x-ray scattering (SAXS) measurements were performed on a SAXSLAB GANESHA 300 XL SAXS system equipped with a GeniX 3D Cu Ultra Low Divergence micro focus sealed tube source producing X-rays with a wavelength of λ = 1.54 Å at a flux of 10^8 photons s^{-1}. The instrument was equipped with a Pilatus 300 K silicon pixel detector. Two configurations with sample-to-detector distance of 713 mm and 1513 mm respectively have been used to access a q-range of $0.015 \le q \le 0.447$ $Å^{-1}$ and $0.007 \le q \le 0.212$ $Å^{-1}$, with wave vector $q = 4\pi(\sin \vartheta/2)/\lambda$. Each sample was measured for 2 hours in each configuration. Silver behenate was used for calibration of the beam center and the q range. Samples were placed in 2 mm quartz capillaries (Hilgenberg, Germany).

The two-dimensional SAXS patterns were brought to an absolute intensity scale using the calibrated detector response function, known sample-to-detector distance, measured incident and transmitted beam intensities, and azimuthally averaged to obtain one-dimensional SAXS profiles. The scattering curves of the sample were obtained by subtraction of the scattering contribution of the solvent and quartz capillaries.

Data was modelled with SasView 3.1.2 (http://www.sasview.org/) using pre-developed form factors for homogeneous,[2] and hollow-cylinders[3]. The solvent electron length density $\rho_{H2O} = 9.47 \cdot 10^{-6}$ have been determined with the SasView dedicated utility.

Static light scattering (SLS) measurements were performed on an ALV CGS-3 instrument equipped with a 532 nm green laser and an ALV-LSE5004 digital correlator. To minimize excluded volume interactions a freshly prepared sample of $[ALV]_3[KGE]_4$ self-assembled by direct dissolution at 0.5 mg/ml in pH 4 buffer has been used. Based on cryoTEM measurements this low concentration does not influence self-assembly behavior compared to assembly at 5-10 mg/ml, see table S3. Analysis was conducted using quartz NMR

tube between 30 and 150 degrees with angular step of 10 degrees. For each angle three runs of 60 seconds have been performed and were averaged over time to obtain the static values $I(q)_{\mathrm{samp}}$, where $q = 4\pi n_{\mathrm{s}}\sin\theta/\lambda$ is the wave vector (with n_s the refractive index of the solvent, θ is half of the scattering angle and λ is the wavelength of the laser). The same procedure has been followed to measure the static intensities of the pH4 $I(q)_{\mathrm{solv}}$ buffer and of a toluene standard $I(q)_{\mathrm{st}}$. These values have been used to bring $I(q)_{\mathrm{samp}}$ on absolute scale (the so-called Rayleigh ratio) according to the relation:

$$(q) = \frac{I(q)_{\mathrm{samp}} - I(q)_{\mathrm{solv}}}{I(q)_{\mathrm{st}}} R_{\mathrm{st}} \frac{n_{\mathrm{s}}^2}{n_{\mathrm{st}}^2}. \tag{1}$$

Here n_{st} and R_{st} are the refractive index and the Rayleigh ratio of the standard (for toluene at 532 nm n_{st} =1.47, $R_{\mathrm{st}} = 2.10 \cdot 10^{-3}$ m-1). The measurements were repeated at different temperatures from 20°C to 80 °C and back to 20°C with a temperature step of 20 °C.

Ultraviolet-visible spectroscopy (UV-vis) measurements were conducted on a Jasco V-650 spectrophotometer using quartz cuvettes. The wavelength was probed between 340 nm and 800 nm with a scanning speed of 100 nm/min, a band width of 1 nm and a data interval of 0.5 nm with a medium response time.

SI2 Support figures

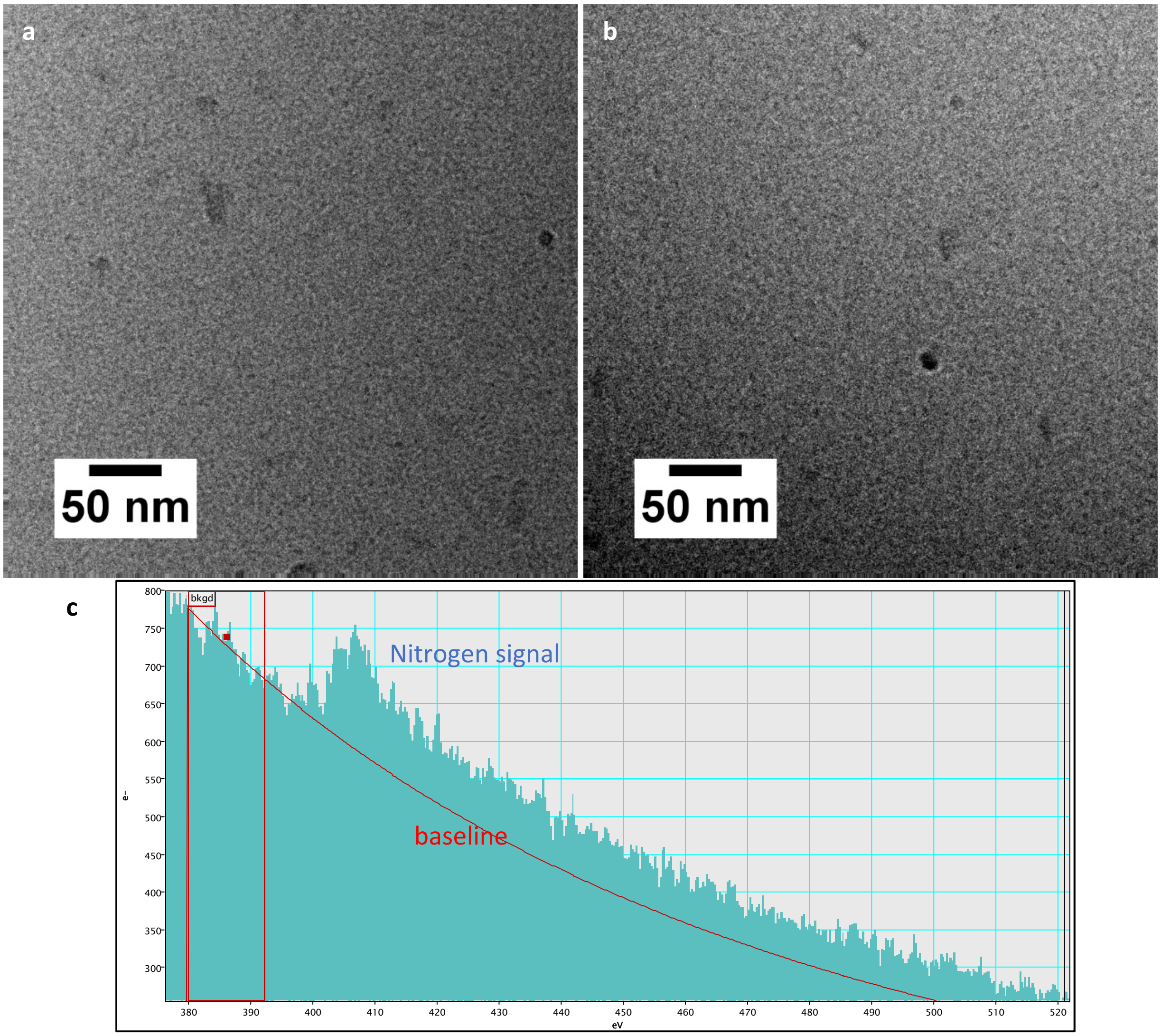

Figure S1 CryoTEM images of $[ALV]_2[KGE]_5$ (a), $r[ALV]_3[KGE]_4$ (b) assembled at 5 mg/ml in pH 4 buffer. Cryo electron energy loss spectroscopy (EELS, c) between 380 and 520 ev of $[ALV]_2[KGE]_5$ in a similar region as figure S1a. The signal at ~400 ev indicates the presence of nitrogen. The only source of nitrogen present in the system is $[ALV]_2[KGE]_5$, confirming its presence in the viewing area.

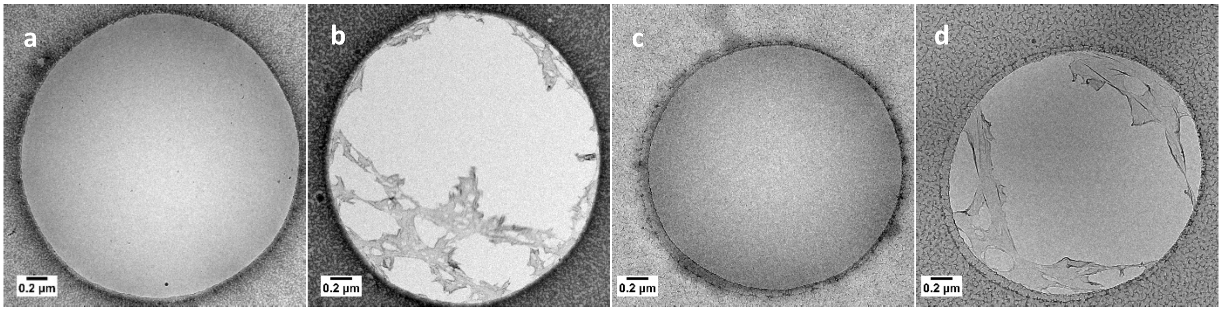

Figure S2 CryoTEM (a,c) and post freeze drying images (b,d) of $[ALV]_2[KGE]_5$ (a,b) and $r[ALV]_3[KGE]_4$ (c,d) assembled in water at 5 mg/ml. If no self-assembled structures are observed during cryoTEM there are two possibilities. Either these structures are removed during blotting or the peptides are present in unimer form. During freeze-drying present peptides crash out of solution resulting in typically dense fibrous networks or sheets that can be observed by TEM.

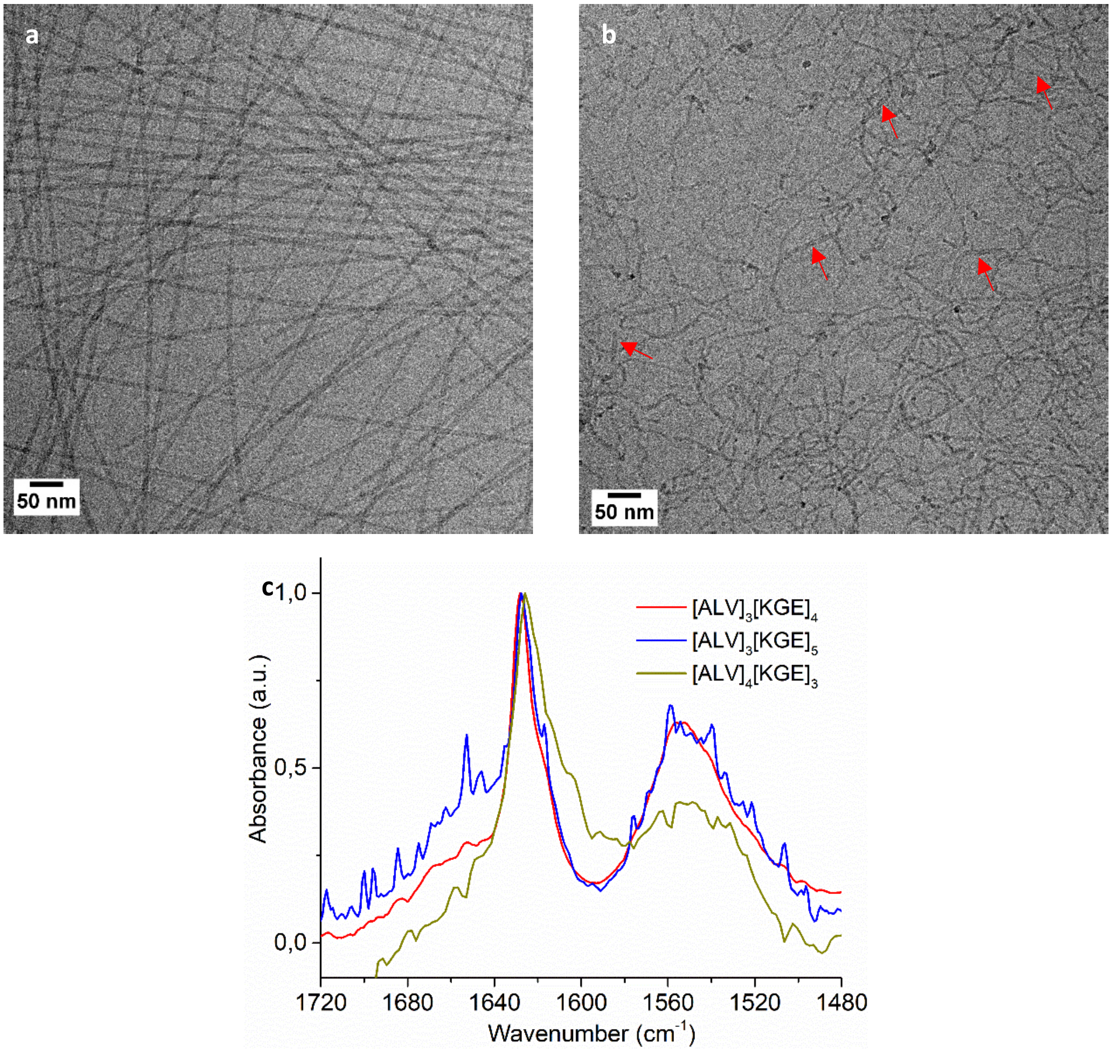

Figure S3 CryoTEM image of $[ALV]_3[KGE]_5$ (a) nanotubes and $[ALV]_4[KGE]_3$ (b) cylindrical shaped micelles and several nanotubes (red arrows) assembled at 5 mg/ml in pH4 buffer. Normalized FTIR spectra of $[ALV]_3[KGE]_5$, $[ALV]_3[KGE]_5$ and $[ALV]_4[KGE]_5$ show strong β-sheet formation for all systems.

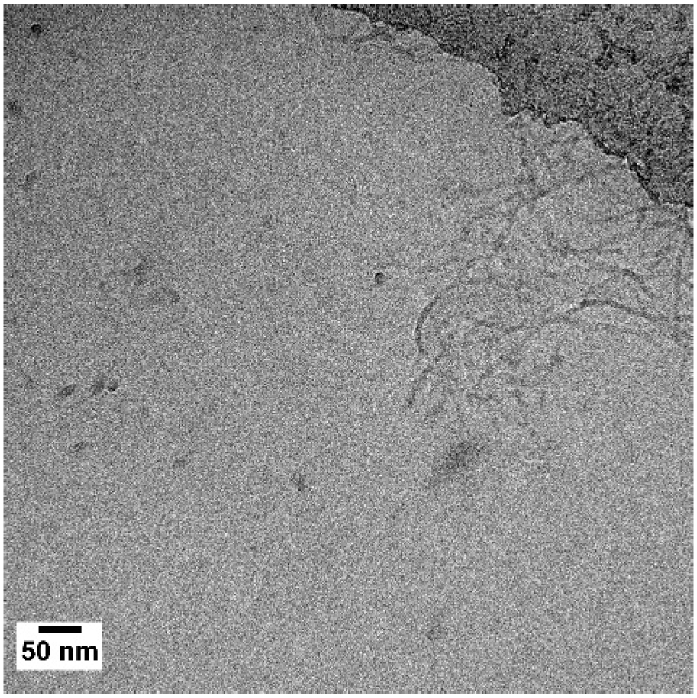

Figure S4 CryoTEM image of observed r[ALV]$_3$[KGE]$_4$ cylindrical micelles assembled at 5 mg/ml in pH4 buffer. This is most likely due to strong self-sorting behavior of the racemic peptide sequences.

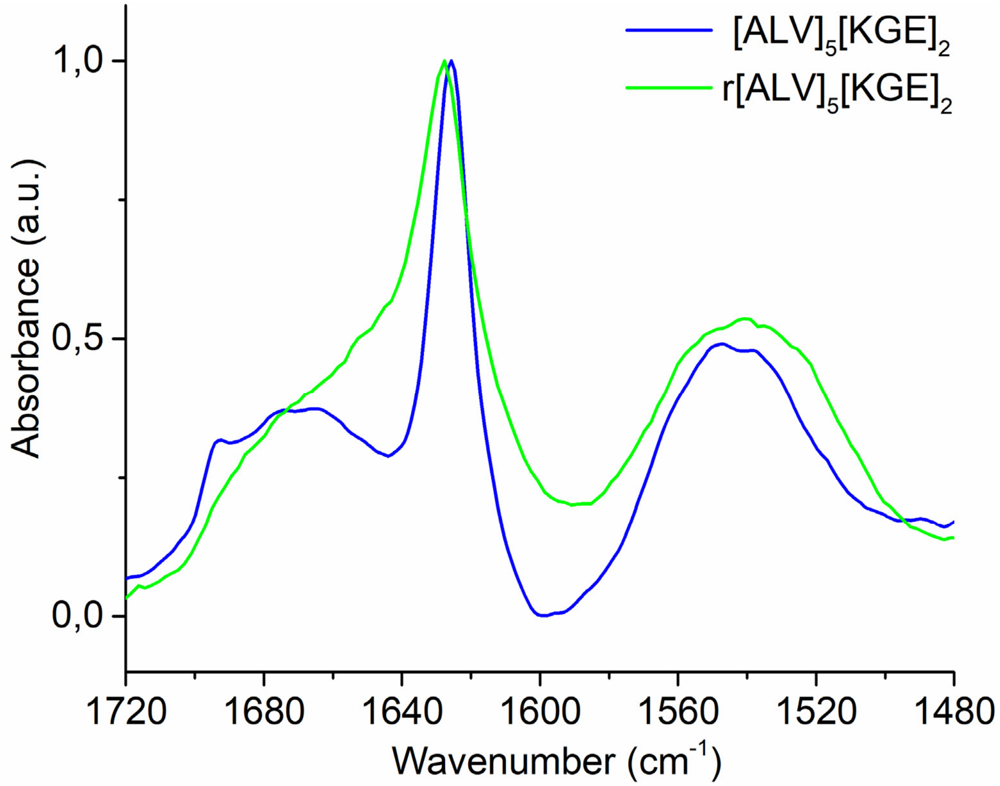

Figure S5 Normalized FTIR spectra of r[ALV]$_5$[KGE]$_2$ and r[ALV]$_5$[KGE]$_2$.

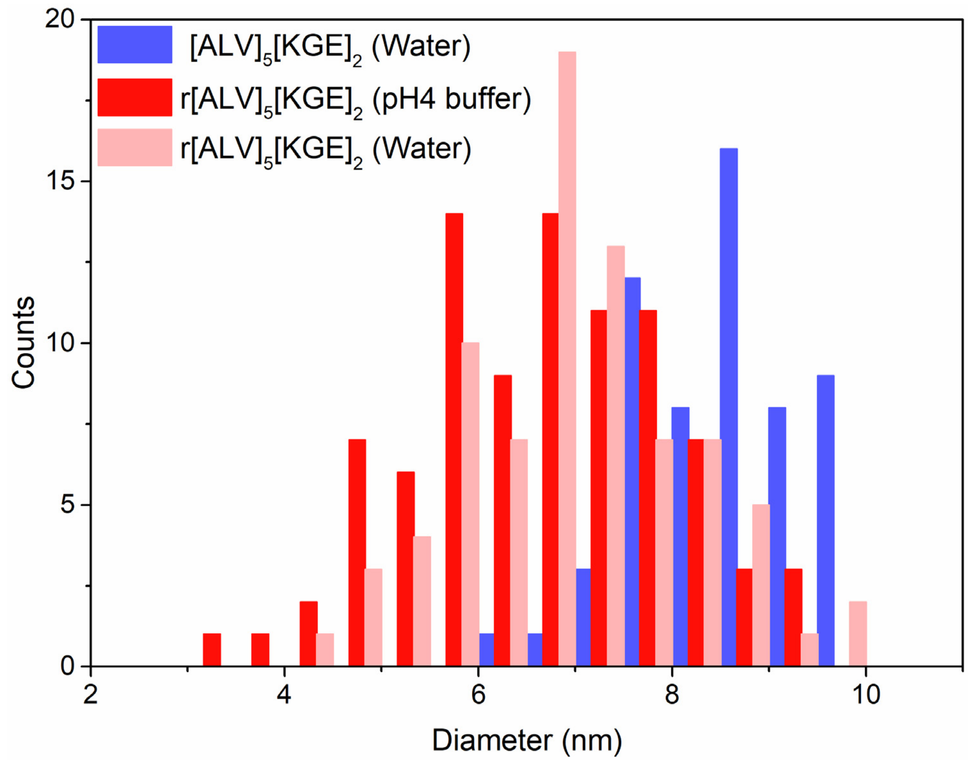

Figure S6 Measured diameter of $[ALV]_5[KGE]_2$ assembled in pure water by a DMSO solvent switch vs $r[ALV]_5[KGE]_2$ cylindrical shaped micelles assembled in pure water and pH 4 buffer via direct dissolution.

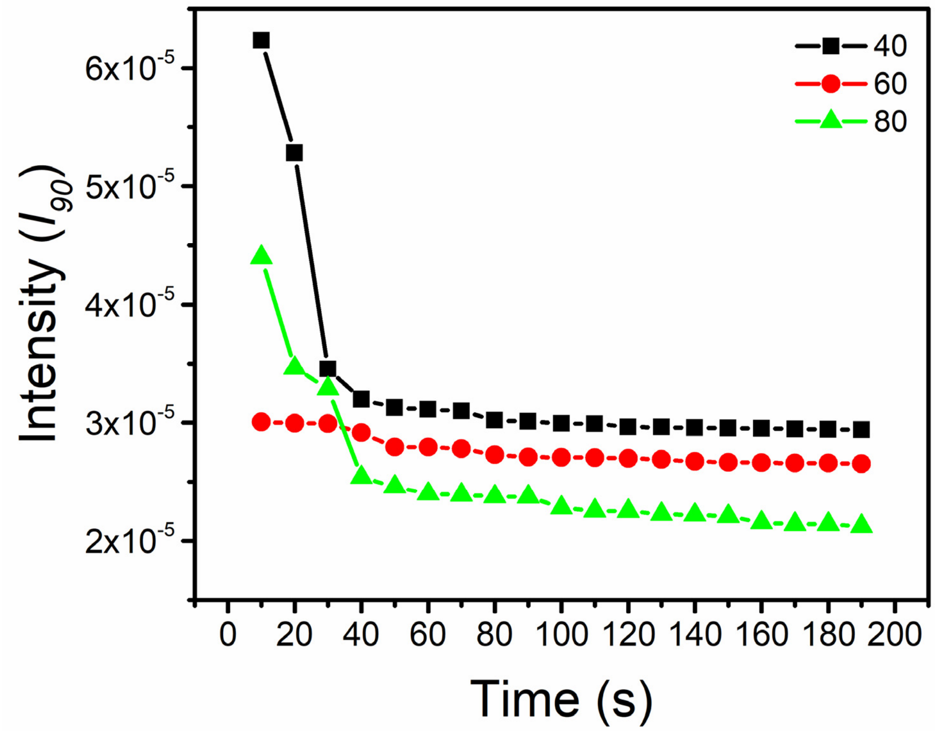

Figure S7 Equilibration time with temperature for each SLS heating step (20-40, 40-60 and 60-80 °C), total scattering intensity measured at a set collection angle at 90 °C.

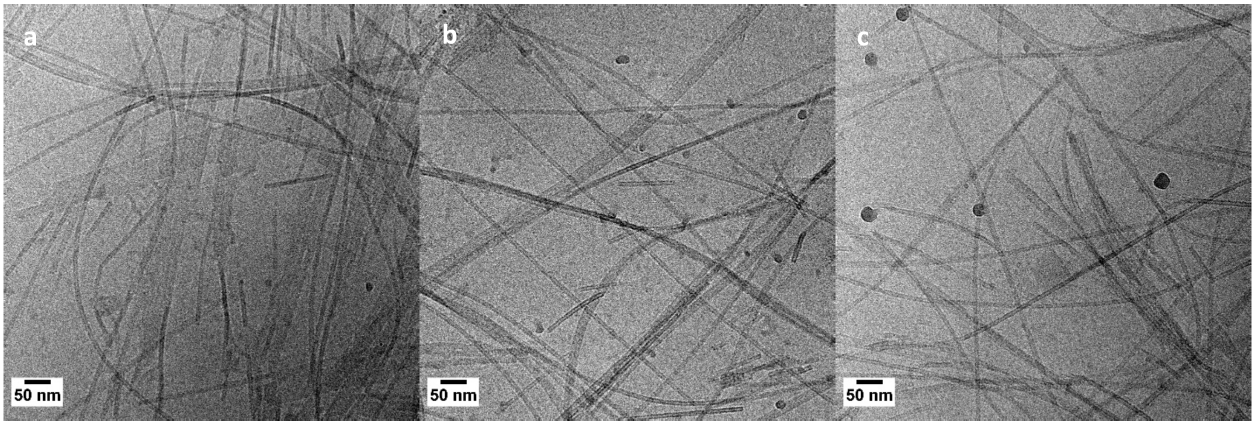

Figure S8 CryoTEM images (a-c) of $[ALV]_3[KGE]_4$ assembled at 0.5 mg/ml heated to 58 °C during vitrification.

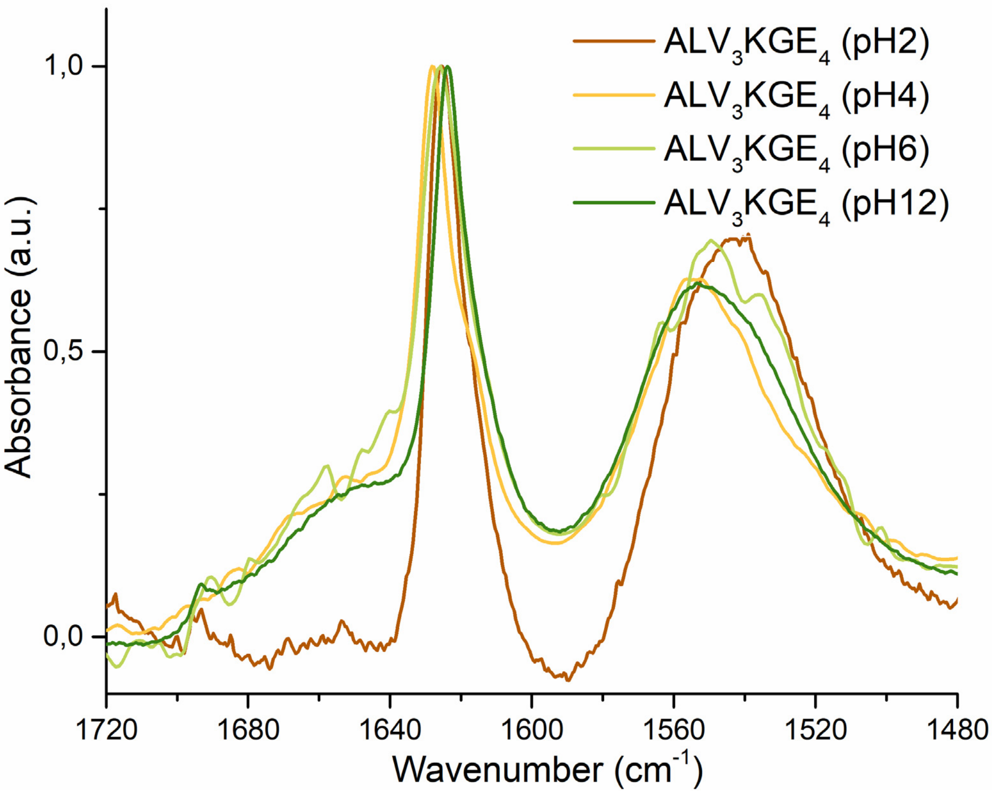

Figure S9 Normalized FTIR spectra of $[ALV]_3[KGE]_4$ assembled at 10 mg/ml in pH 2, 4, 6 and 12 buffer via direct dissolution.

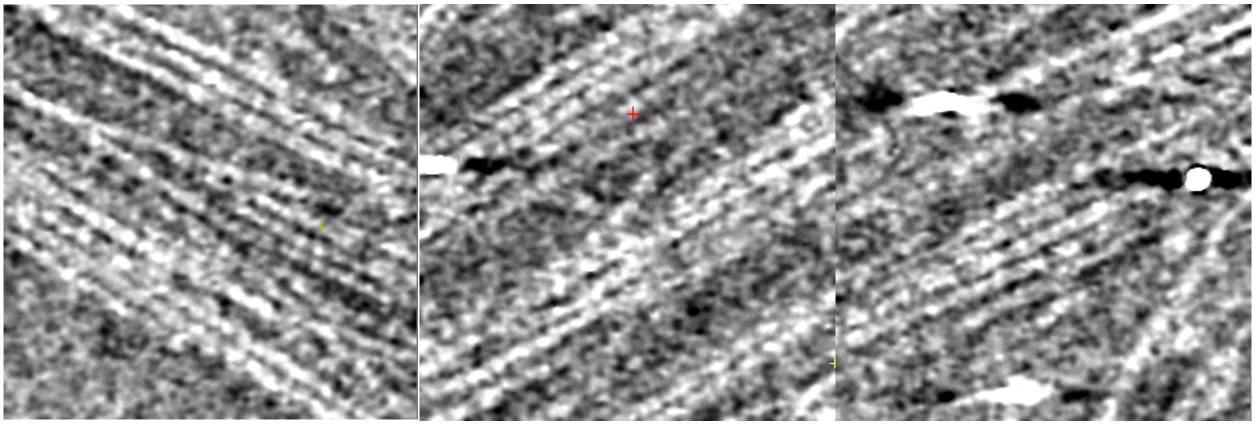

Figure S10 Inverted contrast slices from the cryotomography reconstruction data of $[ALV]_3[KGE]_4$ assembled via direct dissolution at 10 mg/ml in pH 6 buffer.

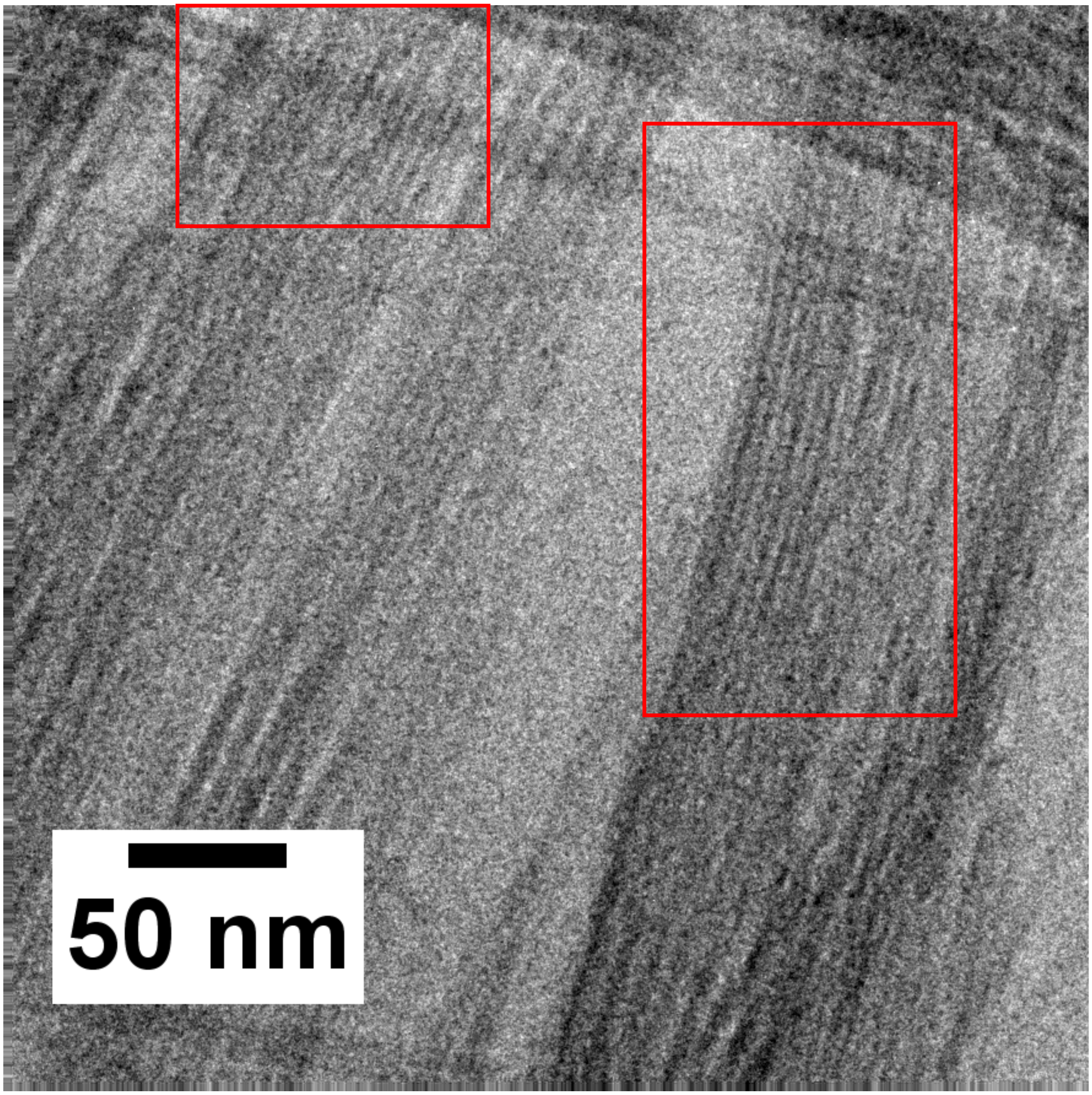

Figure S11 magnification of figure 4e. Regions showing nanotubes with observable the internal cavities are highlighted in red.

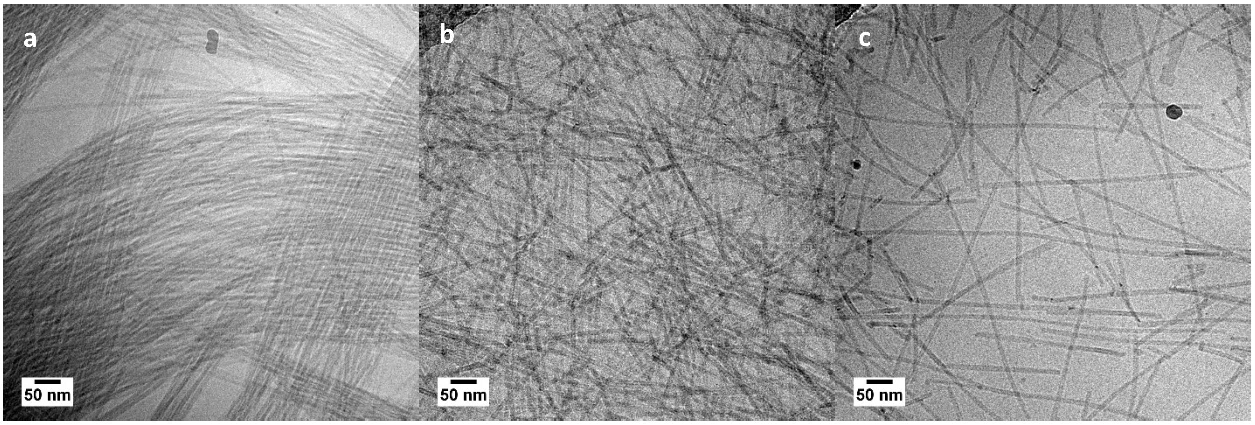

Figure S12 CryoTEM images of $[ALV]_3[KGE]_4$ assembled at 10 mg/ml in pH 6 buffer (a), diluted to 5 mg/ml to pH 6 (b) and diluted to 5 mg/ml at pH 8 (c).

SI3 LC-MS DATA AFTER PURIFICATION

A, [ALV]$_2$[KGE]$_5$ → Ac-ALVALV-b-KGEKGEKGEKGEKGE-NH$_2$

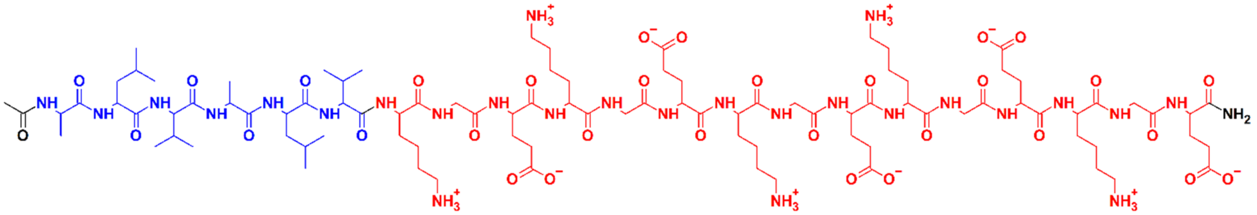

B, [ALV]$_3$[KGE]$_4$ and r[ALV]$_3$[KGE]$_4$ → Ac-ALVALVALV-b-KGEKGEKGEKGE-NH$_2$,

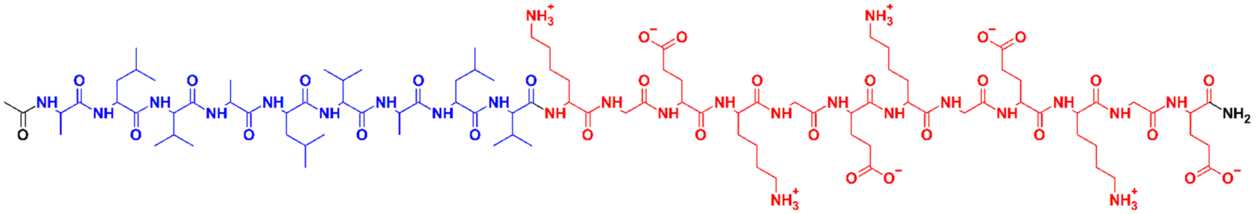

C, [ALV]$_3$[KGE]$_5$ → Ac-ALVALVALV-b-KGEKGEKGEKGEKGE-NH$_2$,

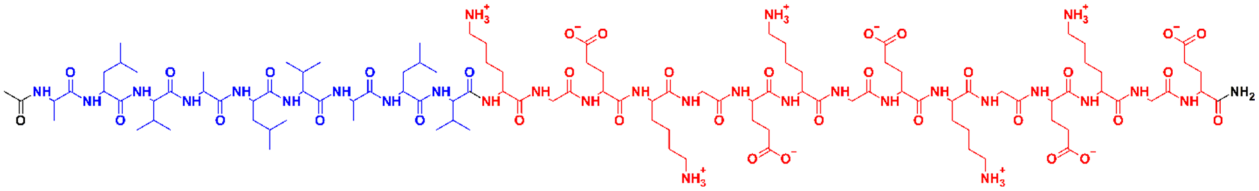

D, [ALV]$_4$[KGE]$_3$ → Ac-ALVALVALVALV-b-KGEKGEKGE-NH$_2$,

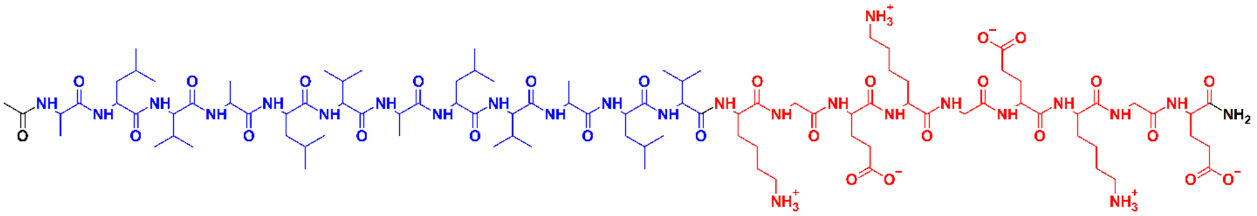

E, [ALV]$_5$[KGE]$_2$ and r[ALV]$_5$[KGE]$_2$ → Ac-ALVALVALVALVALV-b-KGEKGE-NH$_2$,

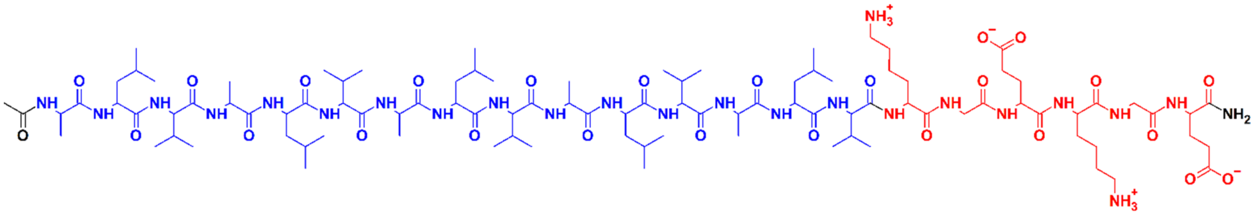

Scheme S1 Molecular formula and chemical structure of the investigated [ALV]$_2$[KGE]$_5$ (a), [ALV]$_3$[KGE]$_4$ (b), [ALV]$_3$[KGE]$_5$ (c), [ALV]$_4$[KGE]$_3$ (d) and [ALV]$_5$[KGE]$_2$ (e) peptide sequences.

Table S2 Expected molar masses of the investigated peptides charged with one, two or three protons.

Peptide	*Expected Mass* *[M + 1H]$^+$*	*Expected Mass* *[M + 2H]$^{2+}$*	*Expected Mass* *[M + 3H]$^{3+}$*
[ALV]$_2$[KGE]$_5$	2198.52	1099.76	733.51
[ALV]$_3$[KGE]$_4$	2167.55	1084.28	723.18
r[ALV]$_3$[KGE]$_4$	2167.55	1084.28	723.18
[ALV]$_3$[KGE]$_5$	2481.89	1241.50	827.96
[ALV]$_4$[KGE]$_3$	2136.58	1068.79	712.86
[ALV]$_5$[KGE]$_2$	2105.61	1053.31	702.54
r[ALV]$_5$[KGE]$_2$	2105.61	1053.31	702.54

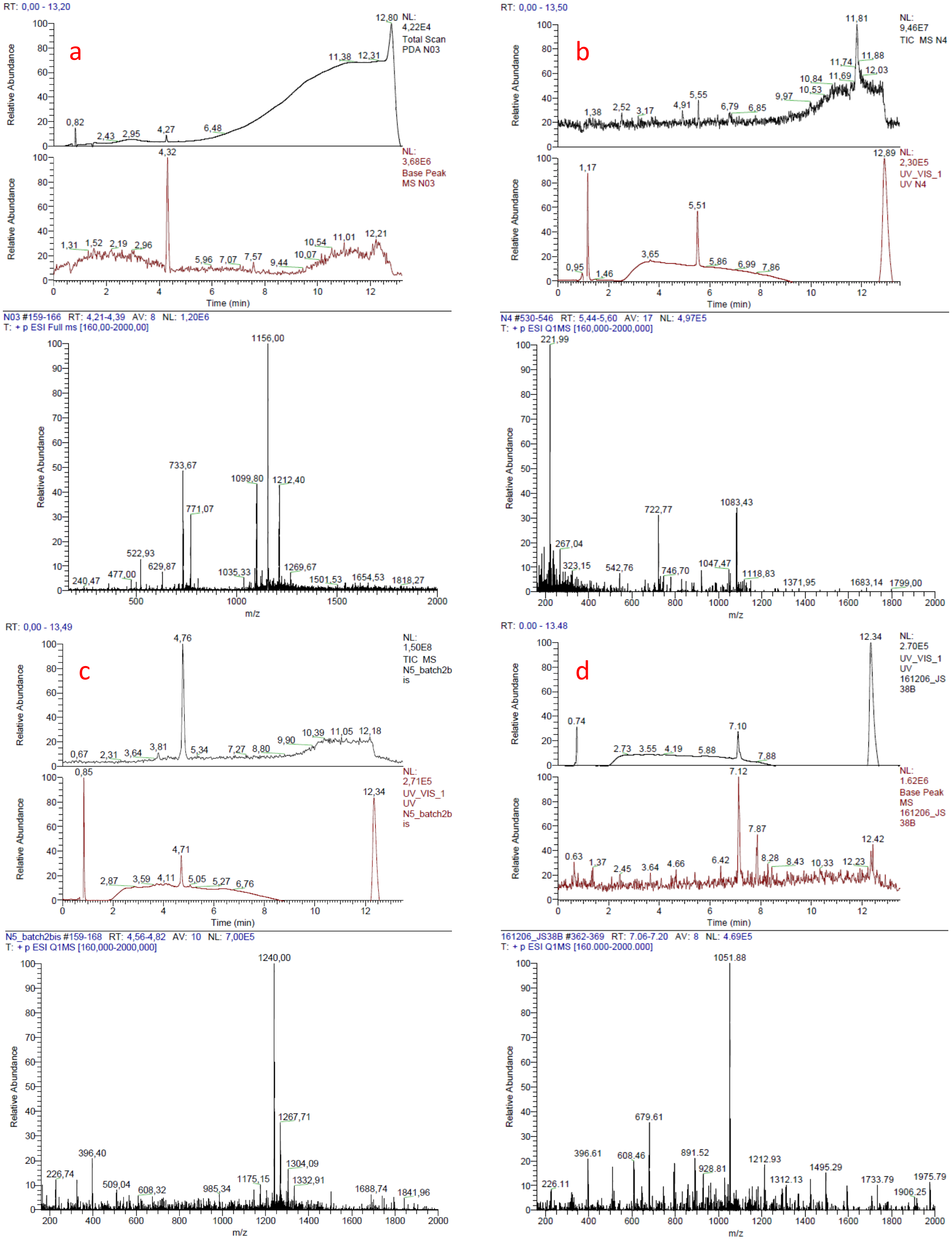

Figure S13 LC-MS spectra of $[ALV]_2[KGE]_5$ with a calculated mass of 2197.52 Da and an observed mass of 1099.80 Da $[M + 2H]^{2+}$ (a), $[ALV]_3[KGE]_4$ with a calculated mass of 2166.55 Da and an observed mass of 1083.43 Da $[M + 2H]^{2+}$ (b), $[ALV]_3[KGE]_5$ with a calculated mass of 2480.89 Da and an observed mass of 1240 Da $[M + 2H]^{2+}$ (c) and $[ALV]_5[KGE]_2$ with a calculated mass of 2104.61 Da and an observed mass of 1051.88 Da $[M + 2H]^{2+}$ (d). $[ALV]_2[KGE]_5$ shows additional TFA adjunct signals 771.07 ((x+TFA)/3), 1156.00 ((x+TFA)/2) and 1212.14 ((x+2TFA)/2). Spectral intensities are normalized for comparison.

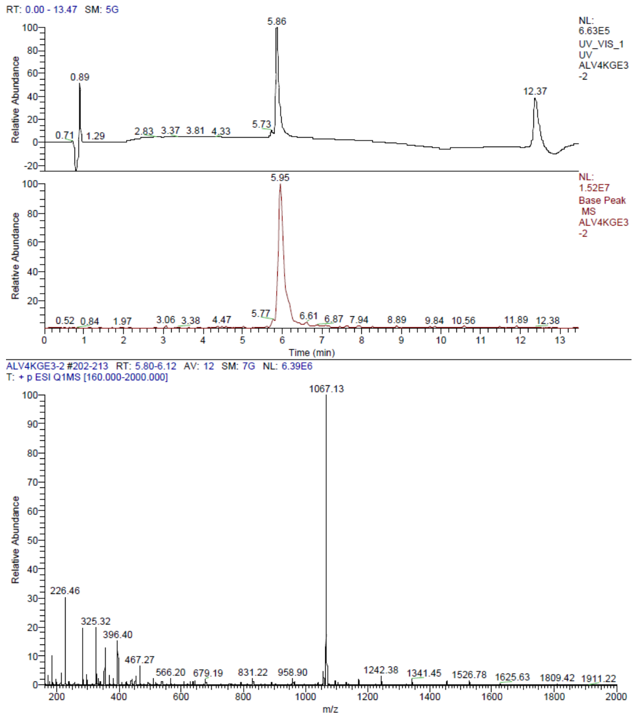

Figure S14 LC-MS spectra of r[ALV]$_4$[KGE]$_3$ with a calculated mass of 2135.6 Da and an observed mass of 1067.13 Da [M + 2H]$^{2+}$. Spectral intensities are normalized for comparison.

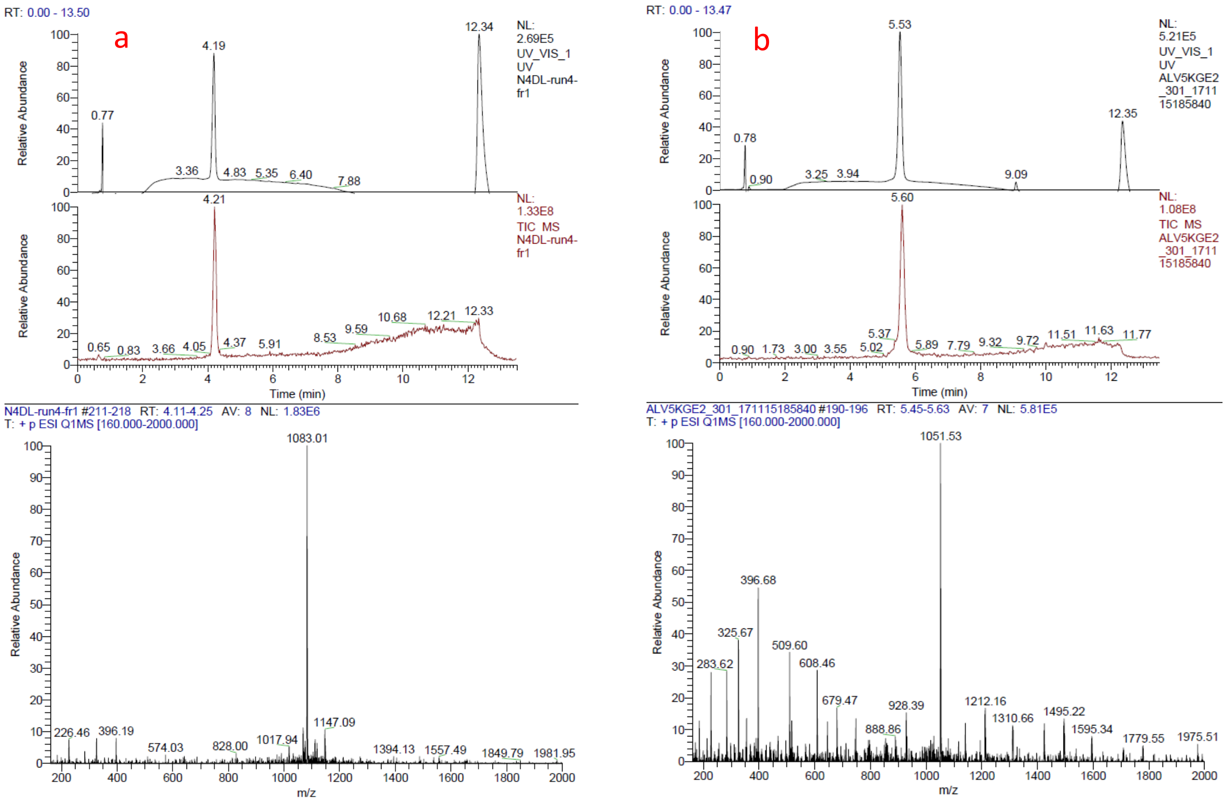

Figure S15 LC-MS spectra of r[ALV]$_3$[KGE]$_4$ with a calculated mass of 2166.5 Da and an observed mass of 1083.01 Da [M + 2H]$^{2+}$ (a) and r[ALV]$_5$[KGE]$_2$ with a calculated mass of 2104.61 Da and an observed mass of 1051.53 Da [M + 2H]$^{2+}$ (b). Spectral intensities are normalized for comparison.

SI4 CRYOTEM OVERVIEW OF ASSEMBLIES

Table S3 Low magnification cryoTEM images of all discussed peptides, assembly- and solution conditions. Blue bolded sample compositions are displayed in figure 1, figure S1 and figure S3.

Peptide	Assembly [mg/ml]	Solvent	CryoTEM Images	
**$[ALV]_2[KGE]_5$**	**DD [10] ****	**Water**	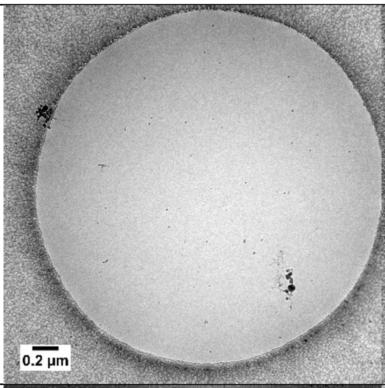	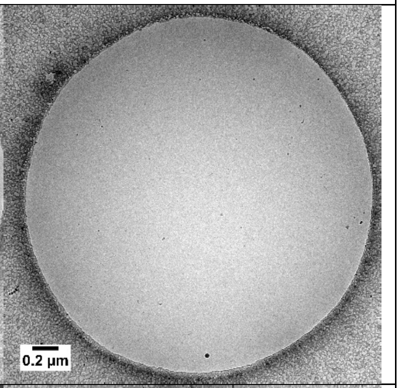
$[ALV]_2[KGE]_5$	DD [10] ***	pH 4 buffer	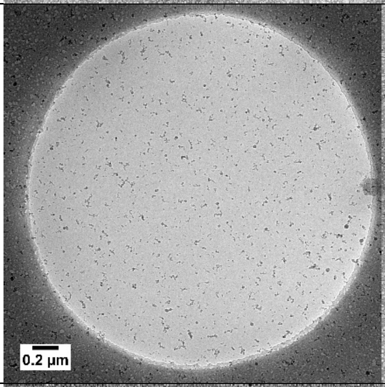	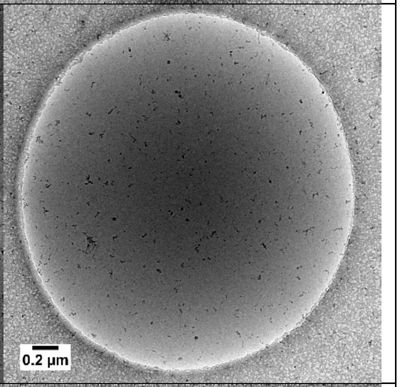
$[ALV]_3[KGE]_4$	DD [10] *	pH 2 buffer	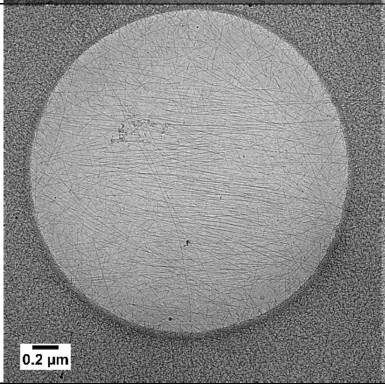	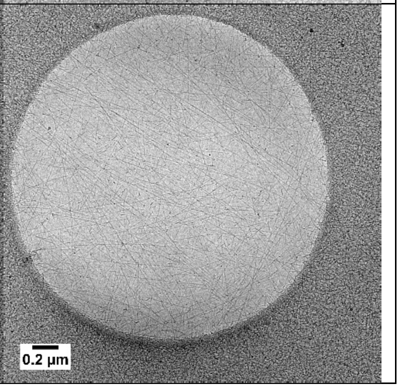

$[ALV]_3[KGE]_4$	DD [10]	pH 4 buffer	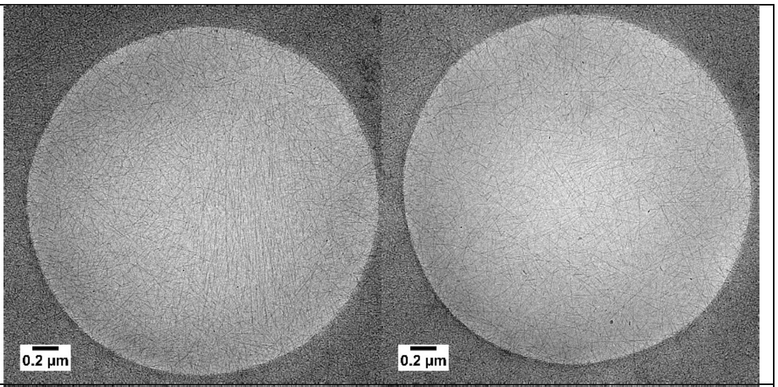
**$[ALV]_3[KGE]_4$**	**DD [5]**	**pH 4 buffer**	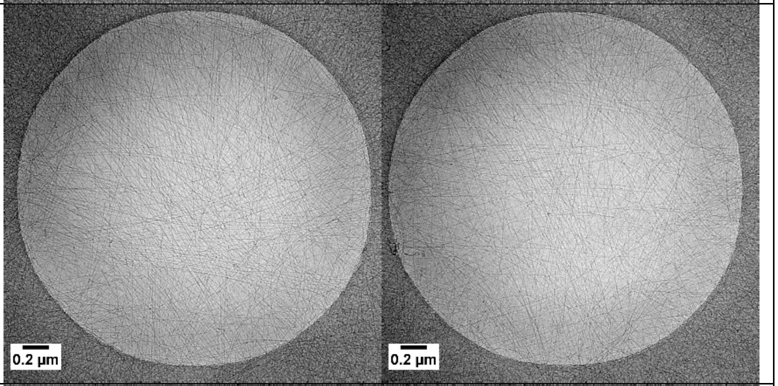
$[ALV]_3[KGE]_4$	SS [5]<br>*	pH 4 buffer	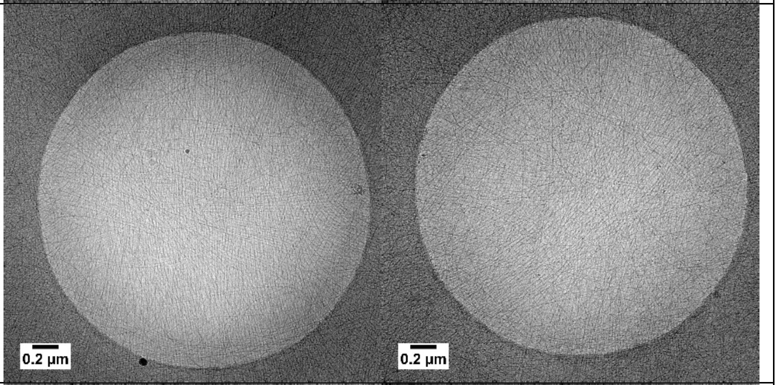
$[ALV]_3[KGE]_4$	DD [0.5]	pH 4 buffer	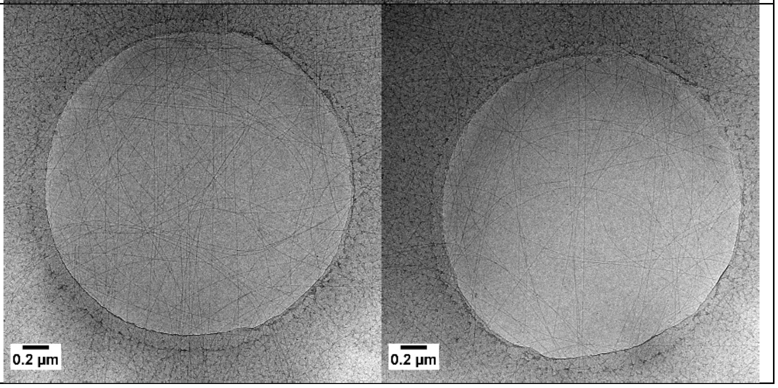

$[ALV]_3[KGE]_4$	DD [10]<br>*	pH 6<br>buffer	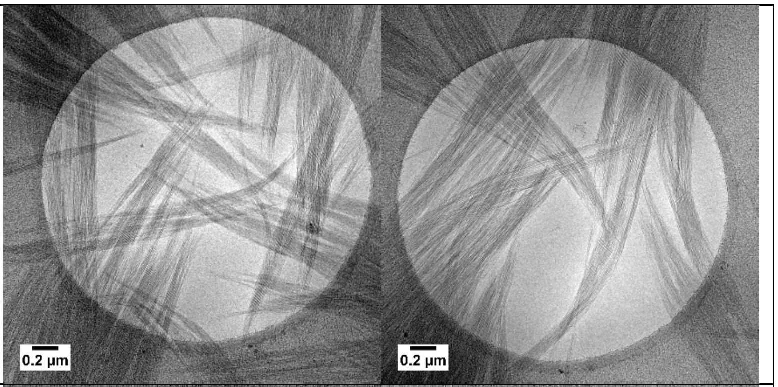
$[ALV]_3[KGE]_4$	DD [1]<br>*	pH 6<br>buffer	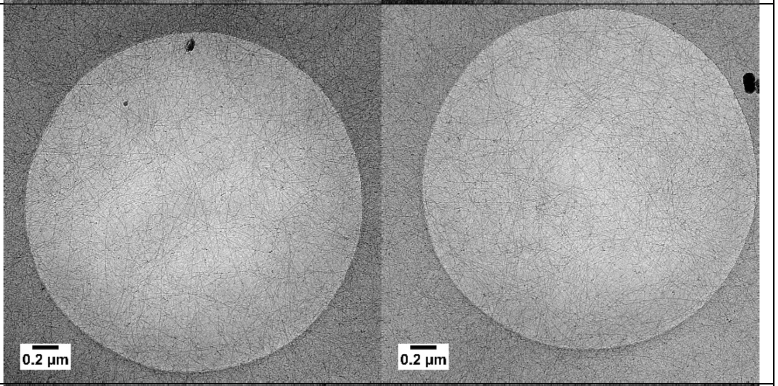
$[ALV]_3[KGE]_4$	DD [10]<br>*	pH 8<br>buffer	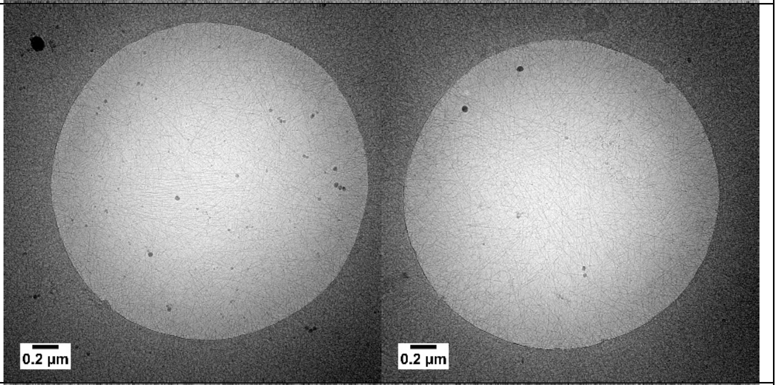
$[ALV]_3[KGE]_4$	DD [10]<br>*	pH 12<br>buffer	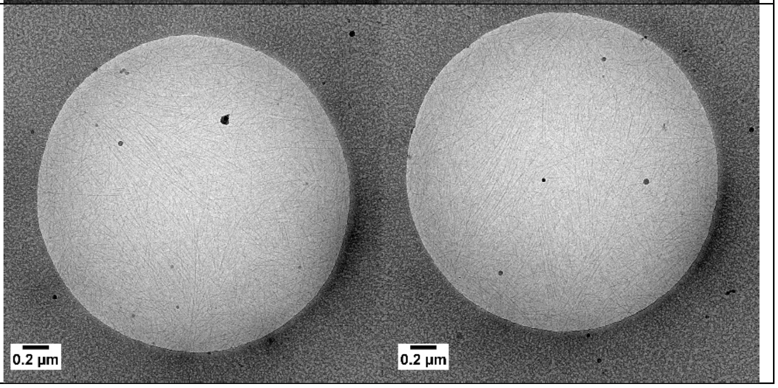

[ALV]$_3$[KGE]$_4$	DD [10]	Water	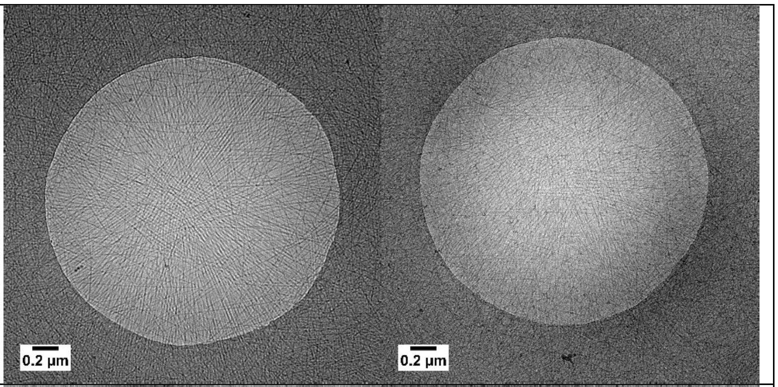
[ALV]$_3$[KGE]$_4$	DD [10]	0.2 M<br>NaCl	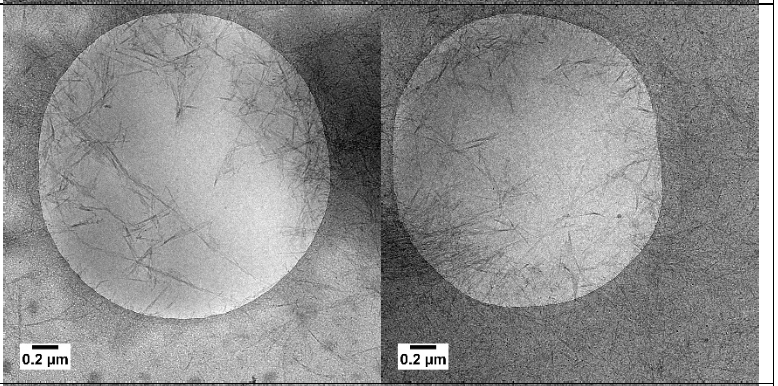
[ALV]$_3$[KGE]$_4$	DD [10]<br>*	8 M Urea	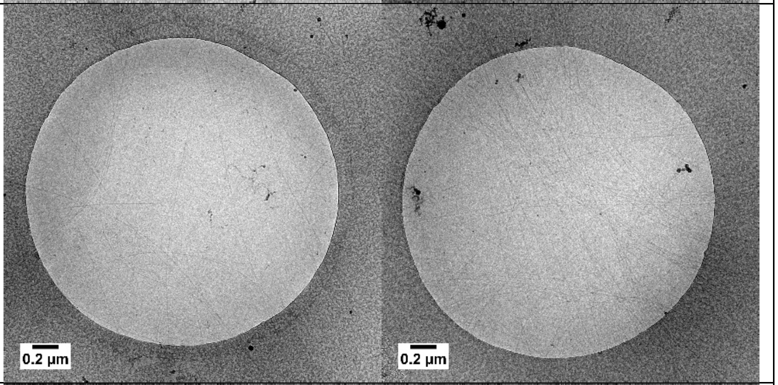
[ALV]$_3$[KGE]$_4$	DD [10]<br>*	3M<br>GdmCl	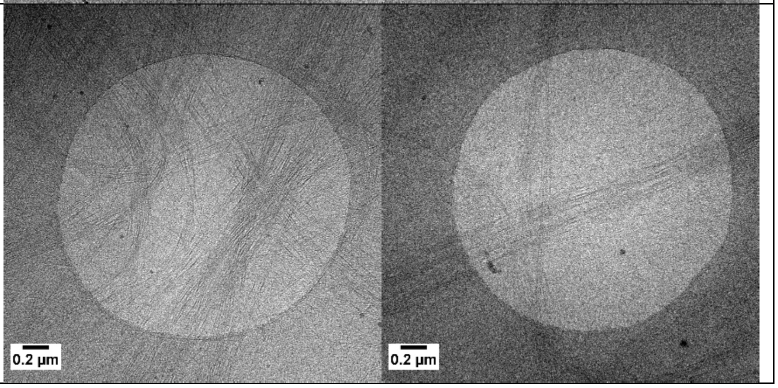

r[ALV]$_3$[KGE]$_4$	DD [5]<br>**	pH 4 buffer	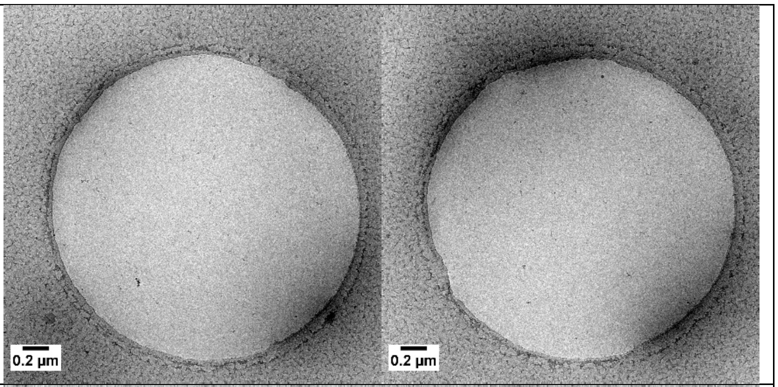
**r[ALV]$_3$[KGE]$_4$**	**DD [5]**	**Water**	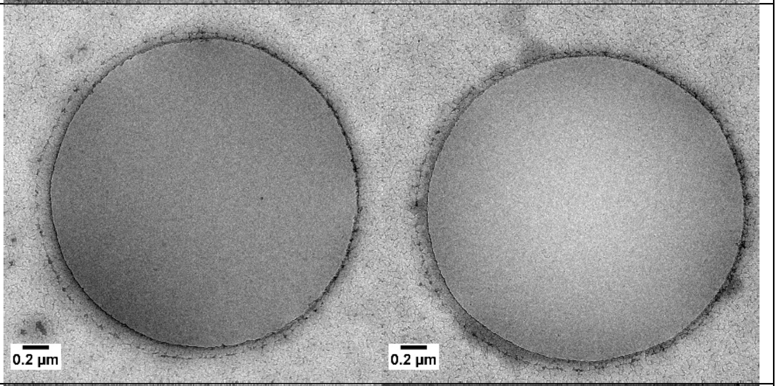
**[ALV]$_3$[KGE]$_5$**	**DD [5]**	**pH 4 buffer**	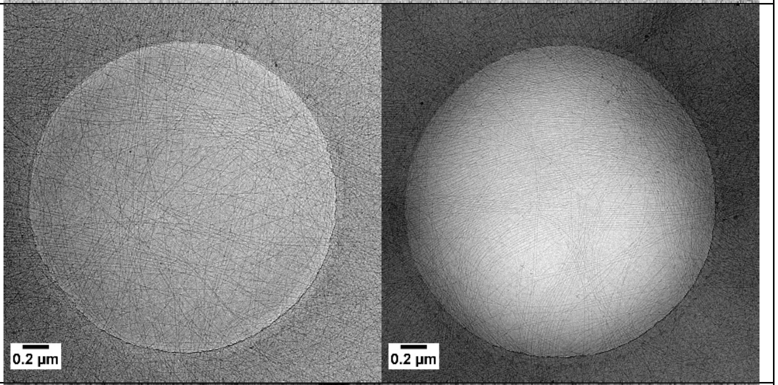
**[ALV]$_4$[KGE]$_3$**	**DD [5]**	**pH 4 buffer**	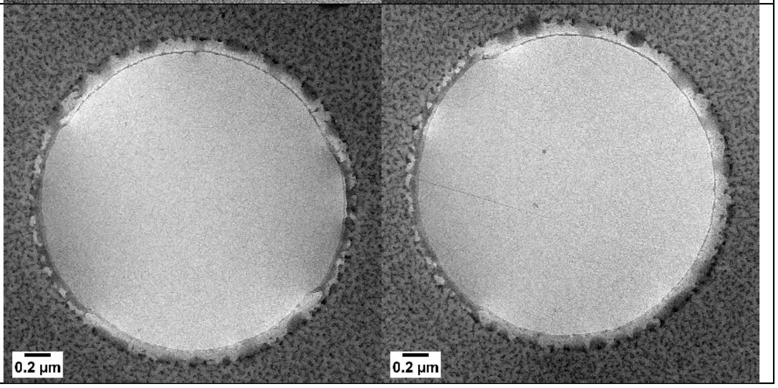

**$[ALV]_5[KGE]_2$**	**SS [10]**	**Water**	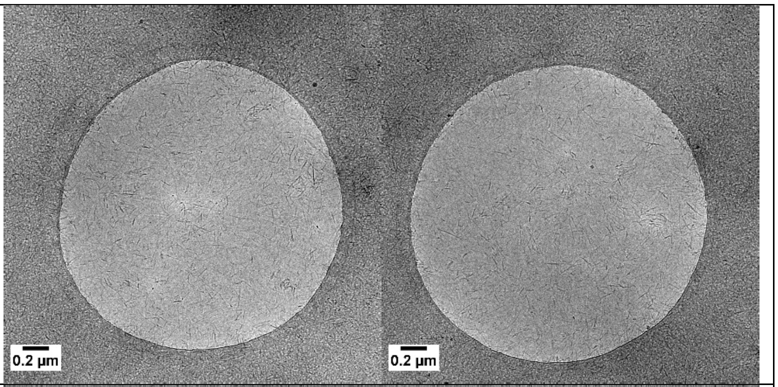
$r[ALV]_5[KGE]_2$	DD [5]	pH 4 buffer	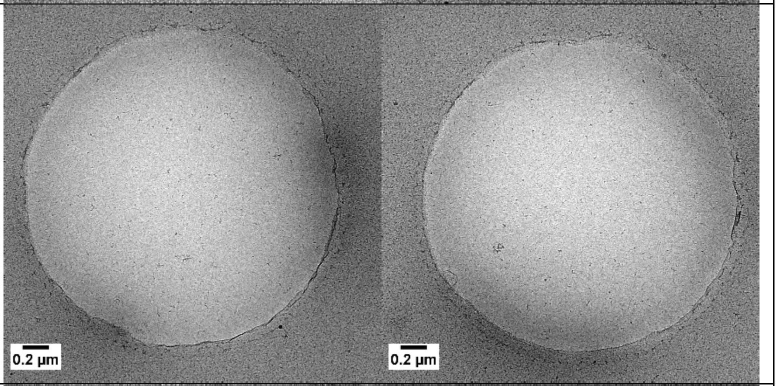
**$r[ALV]_5[KGE]_2$**	**DD [5]**	**Water**	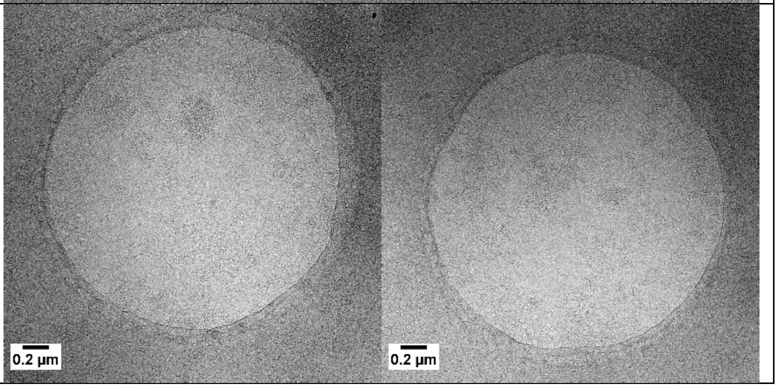

* Incidental ice contamination, sample observed.

** Incidental ice contamination, no sample observed.

*** Large amount of ice contamination, no sample observed.

SI5 DISCUSSION ON NANOTUBE FORMATION

Observance of hollow assemblies by cryoTEM

Most cylindrical structures of $[ALV]_3[KGE]_4$ and $[ALV]_5[KGE]_2$ imaged by cryoTEM show a lower electron transmission at the edge of the cylinder then in the center. This behavior is typical for hollow structures, strongly suggesting that the cylindrical structures are nanotubes, see figure S16. That these typical features for hollow structure are not observed in every assembly and in every cryoTEM image can be explained by three factors; 1) resolution, 2) layer-thickness of the vitrified water and the 3) imaging defocus value.

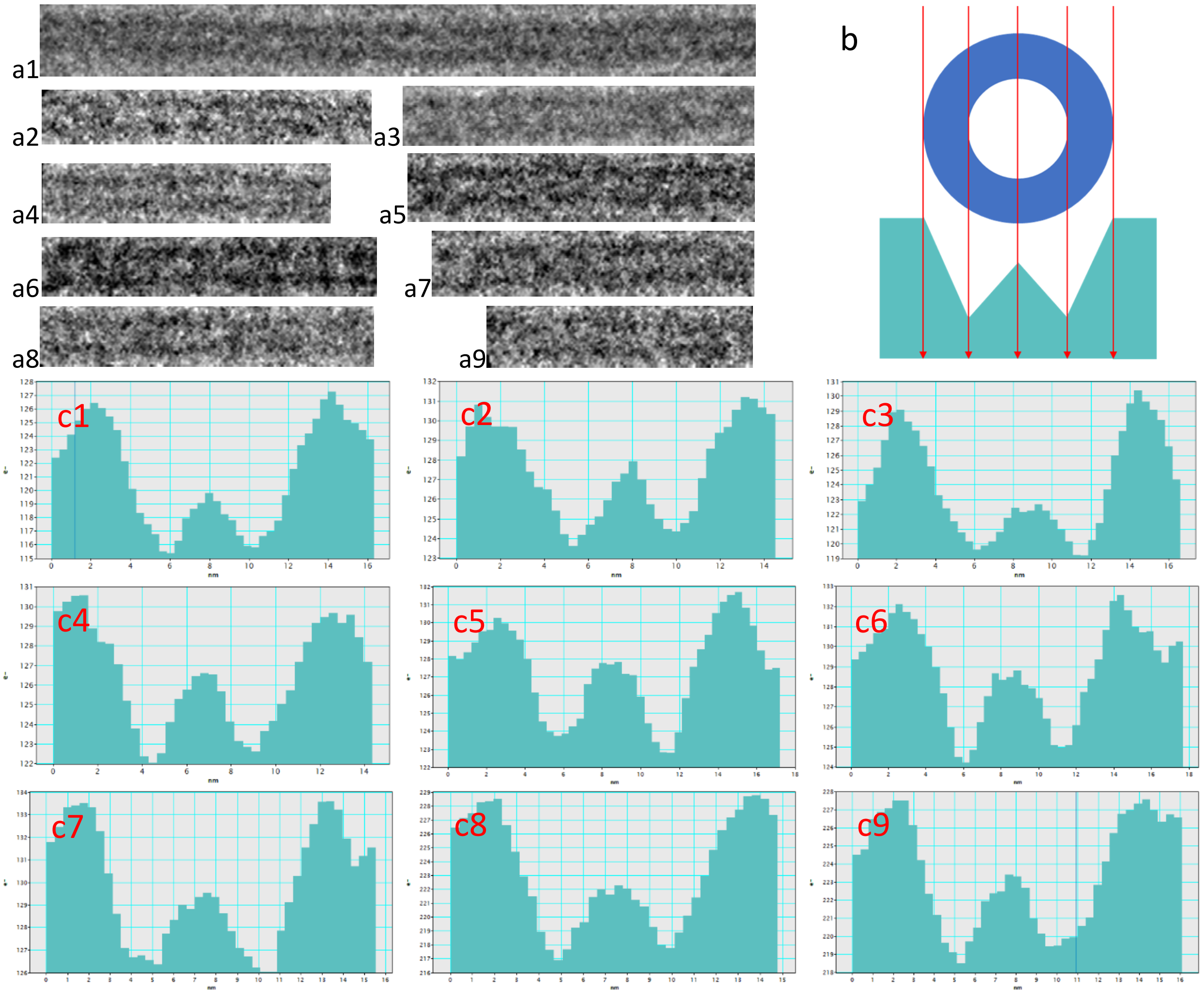

Figure S16 Selected cryoTEM nanotubes from several different $[ALV]_3[KGE]_4$ samples assembled by direct dissolution in pH4 buffer (a1-a9). A schematic of the cross-section and the corresponding transmission signal of a hollow cylindrical object. The line plots, over a length of at least 58.5 nm, of the shown $[ALV]_3[KGE]_4$ nanotubes (c1 – c9).

First, $[ALV]_3[KGE]_4$ nanotubes show an outer diameter of 9 nm and an inner diameter, measured in between the points of lowest intensity, of 4.5±0.4 nm. Under our standard imaging conditions, the pixel size is 0.39 nm. This means that the inner diameter spans 10 - 11 pixels. This minimal number of pixels for the nanotube interior can make it hard to distinguish the wall from the nanotube center. Secondly, in TEM imaging of organic assemblies, contrast is mainly generated due to difference in density between the

object electron path and the electron path of its surrounding. In cryoTEM vitrified water surrounds the sample on all sides, with vitrified water layers ranging from a couple of nanometers to over a micron in thickness. For a nanotube assembly this means that the observed difference in the core and wall contrast would be generated by: the contrast from the wall thickness + the interior water layer + the exterior water layer versus the contrast from the core thickness + the exterior water layer. With the difference in diameter thickness between the peptide nanotube core and the wall of just close to 3 nm the interaction between the electron beam and a thick (200 nm +) vitrified water layers will be dominant. This makes the contrast difference between the peptide nanotube core and the wall difficult to detect. Thirdly, in TEM the resolution and contrast are inversely related depending on the defocus. It was found that at our standard imaging conditions a defocus of close to -4 µm is optimal to observe most features. Below this defocus range the loss in contrast makes it harder to distinguish the assemblies from the environment. Above this defocus value, too much resolution is lost to distinguish between the nanotube core and wall. Because the nanotubes are not perfectly aligned to the grid and multiple layers of nanotubes can be present, therefor there is a difference in height between different nanotubes and segments of individual nanotubes. This difference in height will influence the effective defocus value resulting in the observation of hollow behavior in some nanotubes and regions while not in others.

Peptide molecular configuration

In their native extended state amino acid repeats have a length of ~3.5 nm[4]. This means that the length of a single KGE or ALV repeat is equal to ~1.05 nm. With two C-C bonds (C-C = 1.53 Å)[5] the length of the acetate core block ending is estimated at roughly 3.0 Å. This is an overestimation as it does not take the bond angles nor the presence of half a bond length in the final amino acid repeat into account. On the same bases the amide stabilizer block ending is estimated at 1.5 Å (C-N = 1.46 Å)[5]. Using these chosen values the core, stabilizer and full sequence length of the investigated BCPP can be calculated, *Table S4*. Based on these calculations the maximum diameter of a rod comprising out of two fully extended hydrophobic $[ALV]_x$ blocks is 6.9, 6.9 and 11.1 nm for $[ALV]_3[KGE]_4$, $[ALV]_3[KGE]_5$ and $[ALV]_5[KGE]_2$, respectively. The measured diameter of the assemblies of all these peptides in cryoTEM are 9±1 nm. This means that the peptide core block of both $[ALV]_3[KGE]_4$ and $[ALV]_3[KGE]_4$ is not long enough to assemble into cylindrical micelles. However, they are long enough to assemble in nanotubes with a wall radius of roughly 2 - 3 nm.

Table S4 Formula and calculations for the maximum core, stabilizer and sequence length for $[ALV]_3[KGE]_4$ and $[ALV]_5[KGE]_2$.

Maximum length	Formula	$[ALV]_3[KGE]_4$ (x = 3, y = 4)	$[ALV]_3[KGE]_4$ (x = 5, y = 2)
Core (nm)	L_{core} = 0.30 + x1.05	3.45	5.55
Stabilizer (nm)	$L_{stabilizer}$ =0.15 +y1.05	4.35	2.25
Full sequence (nm)	$L_{sequence} = L_{core} + L_{stabilizer}$	7.80	7.80

Evidence of nanotube formation with SAXS

Two different approaches have been followed to analyze the SAXS patterns: form factor modeling and form factor fitting. The first approach (Figure S17 a) consisted of comparing the experimentally obtained SAXS profiles with the theoretical form factors of a full cylinder having a cross sectional radius of 4.5 nm and the one of a hollow cylinder with an external cross sectional radius of 4.5 nm and an internal radius of 1 nm. In both models the length of the cylinder was set to 400 nm, which is much larger than the larger

length scale accessible experimentally. The baseline and the electron length density of the cylinders have been adjusted manually (no fitting procedure involved) to obtain the best agreement of the curves with the experimental data. The same electron length density value has been used for the solid and the hollow cylinders.

The second approach (Figure S17 b) consisted of fitting the experimental data with the theoretical form factors of full and hollow cylinders respectively. The cylinder length was set as constant (400 nm) while the cross-sectional radius (internal and external in the case of the hollow cylinder), the background and the electron length density of the cylinders have been used as fitting parameters. Cross-sectional Gaussian polydispersity is accounted for in the fitting, with a polydispersity value of 0.08 in both cases. The best fitting was obtained with the form factor of a solid cylinder having cross sectional radius of 4.7 nm and with the form factor of a hollow cylinder having an external cross-sectional radius of 4.5 nm and internal radius of 1 nm.

As expected the main differences between the solid and the hollow models can be observed in the q range associated with typical length scales which are comparable to the cylinder thickness ($0.08 \le q \le 0.2$ Å^{-1} in our case). At lower q values (larger length scales) both models follow the classical scaling power law associated with cylindrical objects $I{\sim}q^{-1}$. The deviation of the experimental data from the theoretical curves at low q might be related with the contribution of the form factor, and is indicative for the presence of repulsive interactions between the cylinders, which might be caused by presence of charge groups at the sides of the cylinders.

In both applied procedures the best agreement with the experimental results was obtained with the hollow cylinder model (Figure S17). Furthermore, the typical sizes obtained by fitting the data with this model are in good agreement with the ones derived from the cryoTEM analysis. Hence the SAXS analysis confirms that the cylinders are hollow.

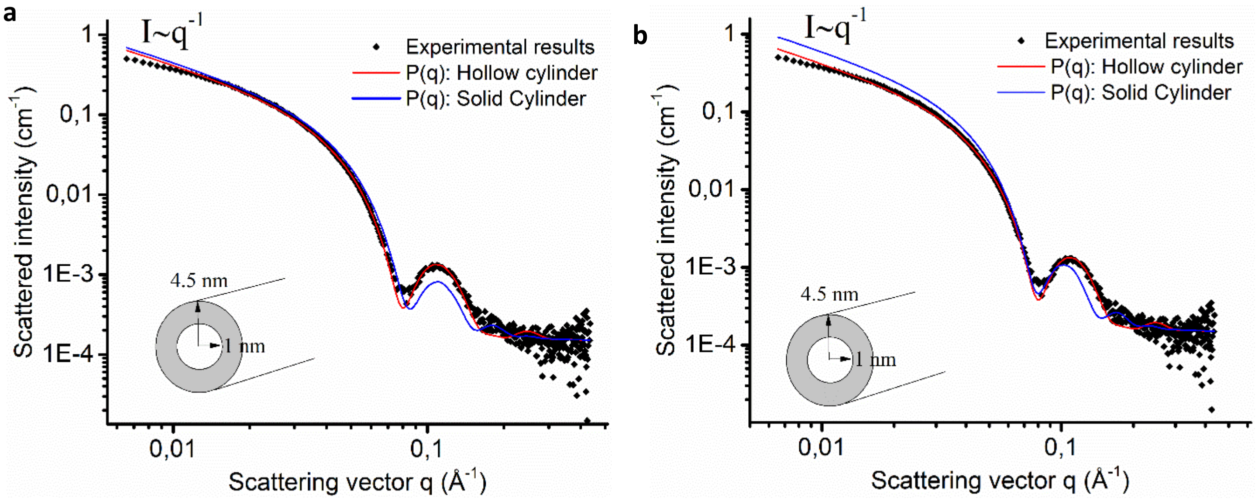

Figure S17 Analysis of the SAXS profiles performed via a) form factor modeling and b) form factor fitting.

SI6 DYNAMIC SELF-ASSEMBLY

To prove unimer exchange between the assemblies and solution, $[ALV]_3[KGE]_4$ was assembled in a pH 4 buffer at 1 mg/ml by direct dissolution. The resulting slightly viscous transparent liquid was dialyzed (10 kDa membrane) for 72 h against 900 ml of the pH 4 buffer. Pre-dialysis cryoTEM confirmed self-assembly into nanotubes (*Figure S18a*). UV-vis analysis (*Figure S18b*) conducted against a pH 4 buffer background, pre-dialysis, showed an absorption maximum at 203 nm of 1.93 Abs, identified as the amide group in $[ALV]_3[KGE]_4$. Post-dialysis UV-vis analysis showed an absorption maxima at 197 nm of 0.25 Abs, indicating a signal decrease of 87 % (1-(0.25-1.93)*100). During dialysis the in-membrane sample volume increased from 0.98 g to 1.19 g, indicating a dilution of only 22 % (=(1.19/0.98)*100-100), which is significantly lower than the observed decrease in absorbance. Therefore, the strong decrease in observed absorbance of the UV maxima suggests a significant decrease in $[ALV]_3[KGE]_4$ peptide concentration after dialysis. 10 kDa pores (2.5 nm in diameter) are too small to fit assemblies with a diameter of 9 nm. However, a single $[ALV]_3[KGE]_4$ molecule would easily transfer through the membrane, suggesting that $[ALV]_3[KGE]_4$ can exchange unimers in water.

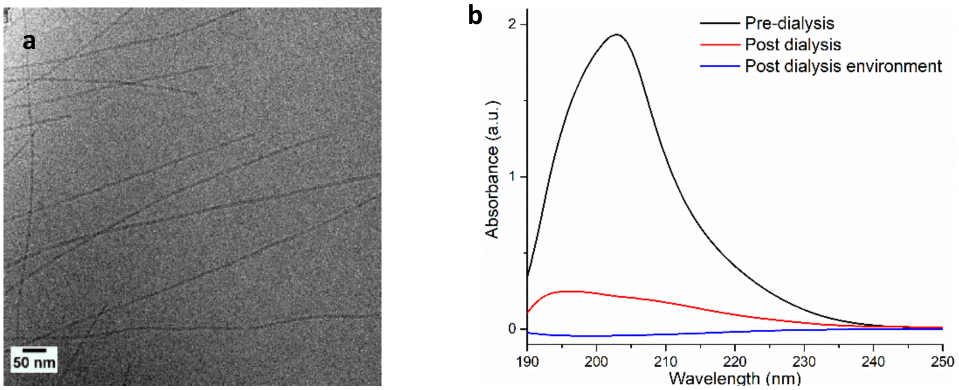

Figure S18 CryoTEM image of [ALV]3[KGE]4 at 1 mg/ml assembled by DD before dialysis (a) and the measured UV spectra (b) of this sample pre-dialysis (black), post dialysis (red) and the outer membrane environment post-dialysis (Blue).

REFERENCES

1. R. A. Robinson and R. H. Stokes, *Electrolyte Solutions: Second Revised Edition*, 1968.
2. A. Guinier and G. Fournet, *Small-Angle Scattering of X-Rays*, John Wiley and Sons, New York, 1955.
3. L. A. Feigin and D. I. Svergun, *Structure Analysis by Small-Angle X-Ray and Neutron Scattering*, Plenum Press, New York, 1987.
4. P. Yurkanis Bruice, *Chem. Eur. J.*, 2007, 1263.
5. G. L. E. Turner, *Ann Sci*, 1991, **48**, 496-497.
